# Detecting warning signs for psychopathology in real time while accounting for context: Two novel statistical process control applications

**DOI:** 10.3758/s13428-026-03004-1

**Published:** 2026-04-21

**Authors:** M. J. Schreuder, E. Schat, E. Ceulemans

**Affiliations:** 1 Quantitative Psychology and Individual Differences, KU Leuven, Tiensestraat 102, 3000 Leuven, Belgium; 2 Department of Developmental Psychology, Tilburg University, Tilburg, The Netherlands; 3 Tilburg Experience Sampling Center, Tilburg University, Tilburg, The Netherlands

**Keywords:** Statistical process control, Experience sampling, Early warning signs, Real-time monitoring, Personalized prediction

## Abstract

Statistical process control (SPC) may detect whether and when repeatedly assessed emotions reach unusual levels, which holds promise for the real-time detection of imminent depression. However, SPC does not account for contextual effects on emotions, such as people feeling systematically worse during stressful events and better during weekends. This may cause false alarms (e.g., presence of warning signs during stressful events) as well as false negatives (e.g., absence of warning signs during weekends). We therefore present two novel context-sensitive SPC methods, which adjust the monitored score according to contextual factors. A simulation study showed that these context-sensitive methods outperform the standard SPC method when contextual effects are large while the effect of depression on emotions is relatively small, but lose their advantage when contextual factors are biased. An empirical illustration confirmed these findings. Context-sensitive SPC methods are thus recommended when contextual factors can be accurately pinpointed, which may hold, for instance, for location and temporal cycles (seasons, menstrual cycles, week/weekend days).

**Supplementary Information:**

The online version contains supplementary material available at 10.3758/s13428-026-03004-1.

The experience sampling method (ESM) allows for studying how momentary emotions, behaviors, and cognitions unfold in individuals’ daily lives (Myin-Germeys et al., 2018). With technological advancements, ESM has become increasingly feasible and accessible, as illustrated by studies that collected repeated daily assessments for several consecutive months or even an entire year (Bos et al., 2022; Helmich et al., 2023; Ludwig et al., 2025; Schreuder et al., 2023; Snippe et al., 2023). Such intensive and long-term sampling has also expanded the potential of ESM. Specifically, it has become possible to monitor within-person changes in the mean level or variability of momentary emotions (Schreuder et al., 2024; Smit & Snippe, 2023; Snippe et al., 2023). As such changes may herald larger transitions—such as recurrent depression—monitoring long-term ESM data may offer significant progress towards the personalized early detection of mental disorders.

A promising method for monitoring ESM data in real time is statistical process control (SPC) (Schat, Tuerlinckx, de Ketelaere et al., 2024a; Smit, Schat, et al., 2023a). SPC consists of two phases of ESM data collection, namely a baseline period (phase I) and a monitoring period (phase II). Phase I should ideally cover a large amount of ESM data (Schat et al., 2023), and is used to compute control limits, which describe the range within which emotions are expected to remain when an individual is healthy and experiences no substantial changes in their mental health (relative to the baseline period). During phase II, newly collected ESM data are compared against the control limits. When an observation exceeds the control limits^1^, it serves as a warning sign indicating an impending change in mental health, which may call for a preventive intervention. Here, two notable strengths deserve attention. First, because control limits and warning signs are defined based on person-specific ESM data, SPC allows for ideographic inferences (i.e., is *this specific person* at increased risk or not). Second, because each new incoming ESM observation can in principle generate a warning sign, SPC allows for real-time inferences (i.e., is this specific person, *at this specific moment in time*, at risk or not). This means that—unlike group-level and retrospective approaches—SPC may yield person-specific warning signs for worsening mental health *before* it has emerged Fig. 1.

**Fig. 1:**
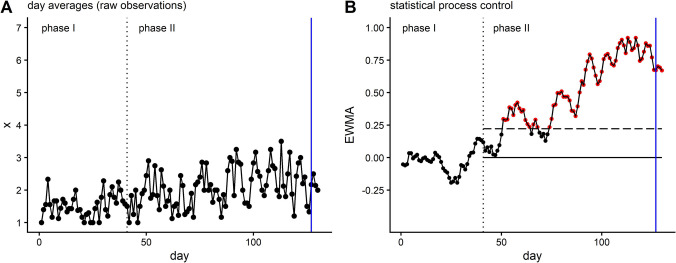
Statistical process control applied to data from a single case study (figure adapted from Schat et al., 2023). **A** Daily averages of restlessness (*x*), which was rated 10 times a day for more than 8 months (Kossakowski et al., 2017). Phase I covers the first 42 days of the study, phase II the remaining days. After 127 days, recurrence of depression occurs (blue vertical line). **B** Statistical process control shows that exponentially weighted moving averages monitored in phase II exceed the control limit (dashed horizontal line), yielding warning signs (red dots) for the recurrent depression

The first empirical studies that applied SPC to detect impending mental health problems yielded carefully optimistic results (Schreuder et al., 2024; Smit & Snippe, 2023; Snippe et al., 2023). Accordingly, SPC has already been used to trigger just-in-time adaptive interventions (Elmer et al., 2025). Whether SPC indeed proves useful in clinical practice, however, depends on the extent to which SPC can be tailored to ESM data. An evident shortcoming in this regard is that SPC does not account for changing contexts. Instead, SPC assumes that control limits remain constant (time-invariant) over time, meaning that emotions are expected to remain within the same (constant) range (Box & Paniagua-Quinones, 2007). Yet, the range of “normal” emotions—i.e., emotions reflective of mental health—likely varies across contexts. To accommodate this, the current study presents and evaluates two novel, context-sensitive methods, which adjust the monitored score according to contextual factors. While such contextual factors may cover many areas, the remainder of this paper will focus on two factors, namely negative events and day-of-week effects (for a rationale, see Supplement S1).

To investigate whether, and to what extent, the accuracy of SPC-based warning signs for impending mental disorders (hereafter, depression) improves when taking into account contextual information (negative events, weekends), we simulated ESM data with daily observations of negative affect (one observation per day). These were affected by negative events, weekends, and depression. Next, we applied the standard (context-insensitive) SPC method (benchmark) as well as two novel SPC methods, which both take into account contextual information (context-sensitive methods). The performance of these methods was evaluated by comparing (1) how quickly warning signs for impending depression were generated^2^ and (2) how late the first false alarms (i.e., warning signs in the absence of depression) emerged. We hypothesized that, especially in the presence of strong contextual effects, context-sensitive methods would perform better than the standard SPC method, which should translate into quicker true alarms and later false alarms. Finally, we applied all three SPC methods to an openly available empirical dataset (Kossakowski et al., 2017) to explore how the simulation study findings translate to real-world scenarios. In what follows, we will describe the standard SPC method, two novel context-sensitive SPC methods, the design of the simulation study that was performed to compare the three methods, and the design of the empirical study that was used to provide an illustrative example of the three SPC methods.

## Methods

We simulated daily ratings of negative affect (NA), denoted as ${y}_{t}$, which depended on contextual factors (negative events, weekends) occurring in both phase I and phase II, depression occurring in phase II, and unobserved random sources of variance (see Simulation procedure). The standard SPC method uses the mean and standard deviation of phase I data (${\widehat{\mu }}_{1}$, ${\widehat{\sigma }}_{1}$) to compute a standardized score that is used for monitoring warning signs. The new, context-sensitive methods adapt this by acknowledging that ${\widehat{\mu }}_{1}$ and ${\widehat{\sigma }}_{1}$ may vary due to contextual factors. Specifically, the modeling method adapts just ${\widehat{\mu }}_{1}$, while the z-method adapts both ${\widehat{\mu }}_{1}$ and ${\widehat{\sigma }}_{1}$, effectively using four different estimates for each parameter (i.e., no event/no weekend; event/no weekend; no event/weekend; event/weekend). Of note, both context-sensitive methods adjust the monitored score, but not the control limit, which thus remains a constant value. Before describing these methods in more detail, we will first elaborate on the standard SPC method.

### Standard statistical process control method

Statistical process control refers to a family of methods, of which the exponentially weighted moving average (EWMA) procedure has been most well investigated in the field of clinical psychology (Schat, Tuerlinckx, de Ketelaere, et al., 2024a; Smit, Schat, et al., 2023a). First, at each time point $t$, negative affect ratings in phase II are standardized by subtracting the mean (${\widehat{\mu }}_{1}$) and dividing by the standard deviation (${\widehat{\sigma }}_{1}$) estimated from the phase I data (formula below). Note that ${\widehat{\mu }}_{1}$ and ${\widehat{\sigma }}_{1}$ are computed over the entire phase I data—without differentiating between different contexts—and hence this method does not incorporate information from contextual factors.

$${z}_{t}=\frac{{y}_{t}-{\widehat{\mu }}_{1}}{{\widehat{\sigma }}_{1}}$$

Next, z-scores are converted into EWMA scores (formula below). Here, $\lambda$ determines the weight of the current z-score (vs. all the previous z-scores), and thus determines the extent to which historical ratings are taken into account. In line with previous work, we will set $\lambda$ to 0.10, which means that current ratings have a relatively small impact on the EWMA score^3^ (Smit, Schat, et al., 2023a, Smit, Snippe et al., 2023b; Schreuder et al., 2024; Smit & Snippe, 2023; Snippe et al., 2023).

$${EWMA}_{t}={EWMA}_{t-1}\times \left(1-\lambda \right)+ {z}_{t}\times \lambda$$

In cases where an EWMA score exceeds the control limit (CL), computed as

$$CL=L\times \sqrt{\frac{\lambda }{2-\lambda }}$$

NA ratings are considered to significantly deviate from phase I. Such a deviation is indicative of a warning sign. Of note, in the one-sided SPC applications considered currently, warning signs occur only when EWMA scores are “too high,” which is based on empirical studies that predominantly focused on monitoring negative emotions prior to recurrent depression. In two-sided SPC applications, warning signs would emerge when EWMA scores are either too high or too low, which could be relevant when the impending change can take opposing shapes (e.g., manic vs. depressive episodes in bipolar disorder). When computing the CL, *L* determines the likelihood of false alarms (i.e., warning signs in the case of stable remission, when the depression effect = 0), and will be set to 2.40. This is in line with previous work, and means that false alarms would be expected to occur only after on average 370 observations (Knoth, 2020; Montgomery, 2009).

The performance of SPC is commonly evaluated based on the timing of the earliest warning sign, averaged across datasets (i.e., the average run length, or ARL). In the case of stable remission (depression effect = 0), ARL values should ideally be high (with *L* = 2.40, around 370), as this would indicate a low probability of false alarms (high specificity). Instead, in the case of a transition towards depression (depression effect > 0), ARL values should ideally be low, as this would indicate a quick detection of recurrent depression (high sensitivity). It follows that ARL curves, which illustrate ARL values for increasing depression effects, should ideally show a steep decline, as this would imply that false alarms are unlikely (high specificity) while true alarms already occur early (high sensitivity)^4^. We will compare the ARL curves obtained with the standard SPC method with those resulting from two novel context-sensitive SPC methods.

### Context-sensitive statistical process control methods

#### Z-method

**Fig. 2:**
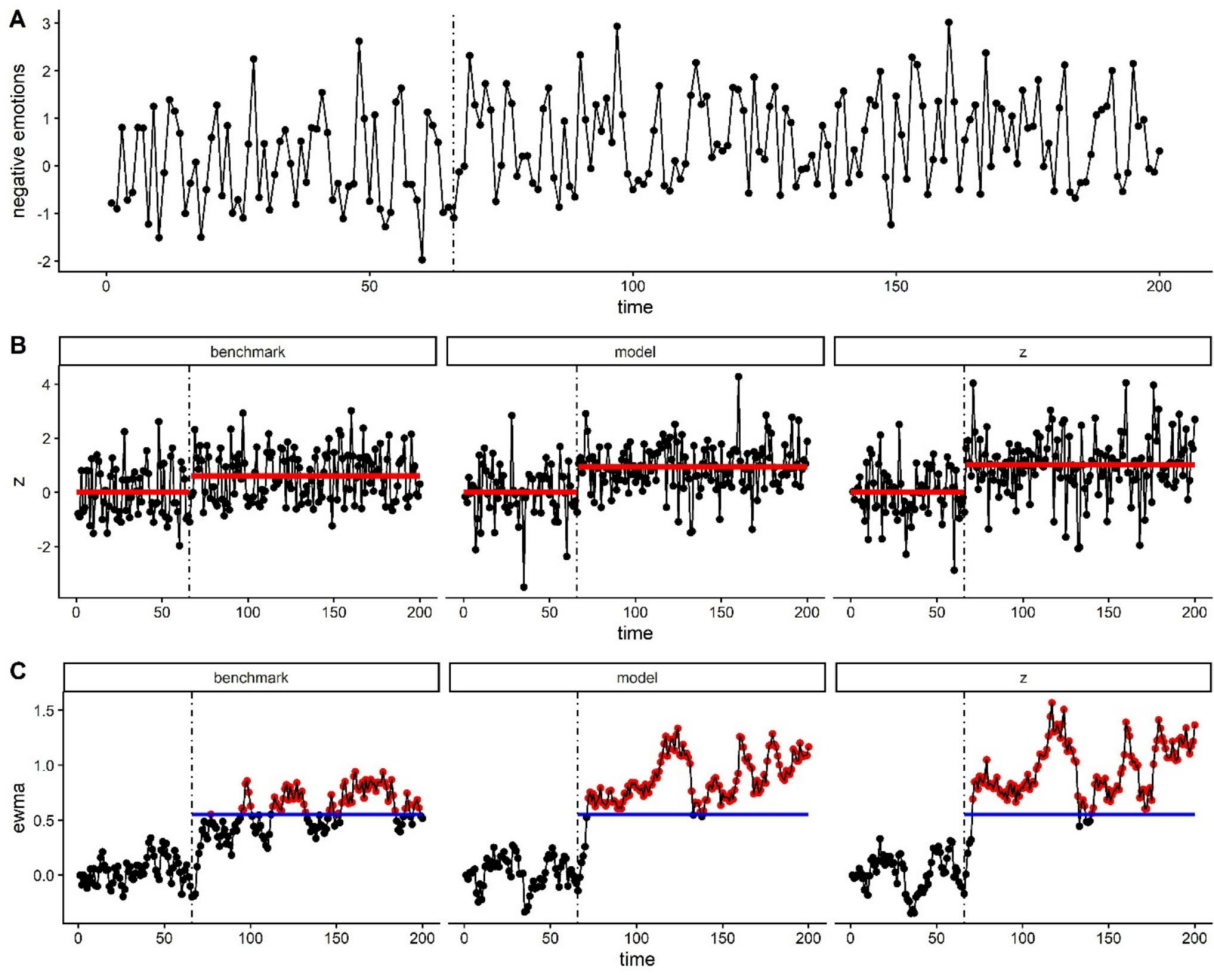
Illustration of simulated ESM data and three different SPC applications (benchmark, model, and z). **A** Raw simulated data, with depression risk (size = 1 SD) at the start of phase II (vertical dotted line). Because of large context effects (events and weekends, both 3 SD for illustrative purposes), the change in mean levels due to depression is hardly visible. **B** When accounting for contextual effects by fitting a linear model (model method) or by adapting the mean and standard deviation used for standardizing scores (z-method), the change in mean levels (horizontal red lines) becomes more pronounced, and thus easier to detect. **C** The EWMA chart for each SPC method, where blue horizontal lines reflect control limits and red dots denote warning signs. For illustrative purposes, contextual effects were exaggerated, providing an advantage for context-sensitive methods in terms of warning sign detection

In the first context-sensitive method (hereafter referred to as the z-method), ${\widehat{\mu}}_{1}$ and ${\widehat{\sigma}}_{1}$ are computed separately based on NA ratings for days with and without events and weekends in phase I. This acknowledges that both the mean and the standard deviation of NA ratings may vary according to contextual factors. Accounting for these contextual factors in the computation of the z-scores means that the effect of other factors (e.g., depression) on NA ratings becomes more prominent, which could improve the performance of SPC. This is illustrated in Fig. 2, which shows that after accounting for contextual variables, the mean-level difference between phase I and II is more pronounced (i.e., the vertical difference between red horizontal lines is larger, Fig. 2B), which in turn means that warning signs are easier to detect (Fig. 2C). The computation of EWMA scores and the control limit remain identical to the standard SPC method, meaning that the monitored score rather than the control limit is adapted.

$${z}_{t}=\left\{\begin{array}{lc}\frac{{y}_{t}-{\widehat{\mu }}_{1\left(00\right)}}{{\widehat{\sigma }}_{1\left(00\right)}}, \ \ \ \ \ \ if \ event=0\ and weekend=0\\ \frac{{y}_{t}-{\widehat{\mu }}_{1(10)}}{{\widehat{\sigma }}_{1(10)}}, \ \ \ \ \ \ if\ event=1\ and\ weekend=0\\ \frac{{y}_{t}-{\widehat{\mu }}_{1(01)}}{{\widehat{\sigma }}_{1(01)}}, \ \ \ \ \ \ if\ event=0\ and\ weekend=1\\ \frac{{y}_{t}-{\widehat{\mu }}_{1(11)}}{{\widehat{\sigma }}_{1(11)}}, \ \ \ \ \ \ if\ event=1\ and\ weekend=1\end{array}\right.$$

#### Modeling method

The second context-sensitive method (hereafter referred to as the modeling method) was inspired by SPC applications in other fields, which handled non-stationarities in the data by first fitting a time series model to the observed data, and subsequently applying SPC to the residuals (Box & Paniagua-Quinones, 2007; de Ketelaere et al., 2011). In line with this approach, we fitted a linear model to phase I NA ratings (${y}_{i}$) with event and weekend as predictors, and used the resulting estimates (${\widehat{\beta}}_{0}$, ${\widehat{\beta}}_{1}$, and ${\widehat{\beta}}_{2}$) to predict phase II data (see formulas below). The residuals from the latter model (i.e., observed NA rating ${y}_{t}$ minus the estimated NA rating ${\widehat{y}}_{t}$) were first standardized and then converted into EWMA scores. Similar to the z-method, the modeling method essentially accounts for variation in NA levels due to contextual factors, thus distilling the effect of depression (Fig. 2B) and potentially improving the performance of SPC (Fig. 2C). Yet, a noteworthy difference between the two methods is that the z-method can in principle account for context-related differences in both mean levels and variability of NA, while the modeling method only accounts for context-related differences in mean levels of NA.

#### Phase I:

$${y}_{i}= {\widehat{\beta}}_{0}+{\widehat{\beta }}_{phaseI,1}\times {event}_{i}+{\widehat{\beta}}_{phaseI,2}\times {weekend}_{i}+{resid}_{i}$$

#### Phase II:

$$\begin{array}{lc}{\widehat{y}}_{t}= {\widehat{\beta }}_{phaseI,0}+{\widehat{\beta}}_{phaseI,1}\times {event}_{t}+{\widehat{\beta }}_{phaseI,2}\times {weekend}_{t}\\ {resid}_{t}= {y}_{t}- {\widehat{y}}_{t}\end{array}$$

### Simulation study

Time series of NA were simulated by first sampling 10,300 NA assessments from a normal distribution (mean = 40, SD = 10), with the first 300 assessments covering phase I and the remaining 10,000 assessments covering phase II (cf. Schat et al., 2023). Subsequently, we added event, weekend, and depression effects. Both negative events and weekends were considered dummy variables with two levels (0 = no, 1 = yes). The extent to which the performance of SPC would improve when adopting context-sensitive methods (vs. the standard method) was expected to depend on the size of the contextual effects. Specifically, in cases where contextual factors have a major impact on NA, accounting for this impact is likely more beneficial than when contextual factors have a negligible impact on NA. We therefore systematically varied the impact of contextual factors. As explained below, the ranges of effect sizes of negative events, weekends, and depression risk on NA were based on empirical research^5^.

#### Negative events

Earlier studies reported that negative events may increase NA by around 0.5 (Kraiss et al., 2024; Machell et al., 2015; Sheets & Armey, 2020; Van Der Stouwe et al., 2019; Von Klipstein et al., 2023) or 1 SD (Anderson et al., 2021; Bai et al., 2020; Booij et al., 2018; Gunthert et al., 2005; Lamers et al., 2018; Peeters et al., 2003). Some studies reported even larger effects (Booij et al., 2018; Drake et al., 2022; Thompson et al., 2012; Zhaoyang et al., 2020), and we therefore used effect sizes of 0.5, 1, and 1.5 SD, thus implying an average NA increase of 5, 10, and 15, respectively. We further varied the frequency of negative events to either one-third or one-fifth of measurement occasions, which is in line with empirical studies showing that people report negative events on 14–25% (Lamers et al., 2018; Nelson et al., 2018; Schricker et al., 2023; Sheets & Armey, 2020) or over 40% (Almeida, 2005; Charles et al., 2013; Von Klipstein et al., 2023) of measurement occasions. Of note, in empirical settings, it may be difficult to determine (with certainty) whether or not negative events have occurred^6^. Such uncertainty could affect the performance of context-sensitive procedures, and we therefore systematically varied uncertainty concerning the occurrence of negative events. This was done by randomly altering 0%, 10%, 20%, and 30% of the binary event occurrence scores in phase II, meaning that 0s were replaced by 1s and vice versa. The effect of this event uncertainty (later referred to as bias) on the performance of the context-sensitive procedures could give an indication of the robustness of these procedures.

#### Weekends

Regarding the effect of weekends on negative affect, earlier studies showed that negative affect is around 0.4 SD lower on weekends than on weekdays (Díaz-Morales & Parra-Robledo, 2021; Hülsheger et al., 2022). Smaller (Areni et al., 2011; Ryan et al., 2010; Stone et al., 2012) and larger effects (Harvey et al., 2015; Helliwell & Wang, 2015) have also been reported, and we therefore used effect sizes of 0.15, 0.4, and 0.65 SD, thus implying that weekends reduce NA levels by 1.5, 4, or 6.5 points, respectively. Further, it is possible that weekends alter not only mean levels of NA but also the variability of NA, for instance because weekends may come with less routine and more out-of-the-ordinary experiences. To examine the impact of such heightened variability, we systematically varied whether or not normally distributed noise (mean = 0, SD = 5) was added on weekend days^7^. This had a small effect on the overall distribution of NA scores (SD change from 11.5 to 11.8) and a larger effect on the distribution of NA scores during weekends (SD change from 11.1 to 12.1).

#### Depression

Depression risk was assumed to occur suddenly from the start of phase II onwards, and was modeled as a dummy variable (0 in phase I, 1 in phase II). Effect sizes ranged between 0 SD (no depression risk, or stable remission) and 1.5 SD (severe depression risk) in steps of 0.25. This range of effects is in line with earlier studies (Schat et al., 2023; Schat, Tuerlinckx, Schreuder et al., 2024b) and corresponds to the finding that differences in mean NA levels between healthy and depressed samples usually exceed 1 SD (Ben-Zeev et al., 2009; Bouwmans et al., 2017; Bylsma et al., 2011; De Calheiros Velozo et al., 2023).

In total, the simulation involved 1,008 design cells (24 combinations of negative event effects, 6 combinations of weekend effects, 7 depression effects), each including 100,000 datasets, amounting to more than 100 million datasets that were all analyzed three times, using the standard SPC method, in which contextual factors were not taken into account, and the two context-sensitive SPC methods (z, model). In line with our hypotheses, we expected that context-sensitive methods would yield steeper ARL curves than the standard method, and that these differences would become more apparent with larger effects of contextual factors.

#### Illustrative empirical case study

In order to explore whether the inferences drawn from the simulation study would also hold in empirical data, we reanalyzed ESM data from an openly available case study (Kossakowski et al., 2017). This study included an adult male with a history of major depressive disorder, who took part in an ESM study with 10 assessments per day for almost 8 months (Kossakowski et al., 2017; Wichers et al., 2016). During this period, he tapered his antidepressant medication, which is a known risk factor for experiencing a relapse. This relapse indeed occurred after around 4 months (day 127). A unique feature of the case study is that it covers a baseline ESM period (i.e., first 41 days, during which tapering had not yet started) as well as clear relapse of depression. This makes the case study ideal for investigating whether SPC could detect warning signs—namely, significant changes in the participant’s momentary mental states—preceding the relapse of depression (Schat et al., 2023; Schat, Tuerlinckx, de Ketelaere, et al., 2024a; Smit, Schat et al., 2023a). For the current purposes, data from the case study were reanalyzed using the three different SPC methods to validate findings from the simulation study. Specifically, we monitored changes in day-averages of 12 mental states (down, irritated, lonely, anxious, suspicious, guilty, doubt, restlessness, agitated, worrying, shame, self-doubt). Some of these mental states are commonly reported in prodromal phases of depression (e.g., restlessness, agitation), while others may be more prevalent at later stages, and thus would be expected to have longer run lengths. Negative events were defined as events that were rated as unpleasant (i.e., score < 0 on a scale from −3 to 3), which occurred on around 18% of measurement occasions. Methodological details regarding the setup of the study are available elsewhere (Kossakowski et al., 2017; Wichers et al., 2016).

## Results

### Simulation study

**Fig. 3:**
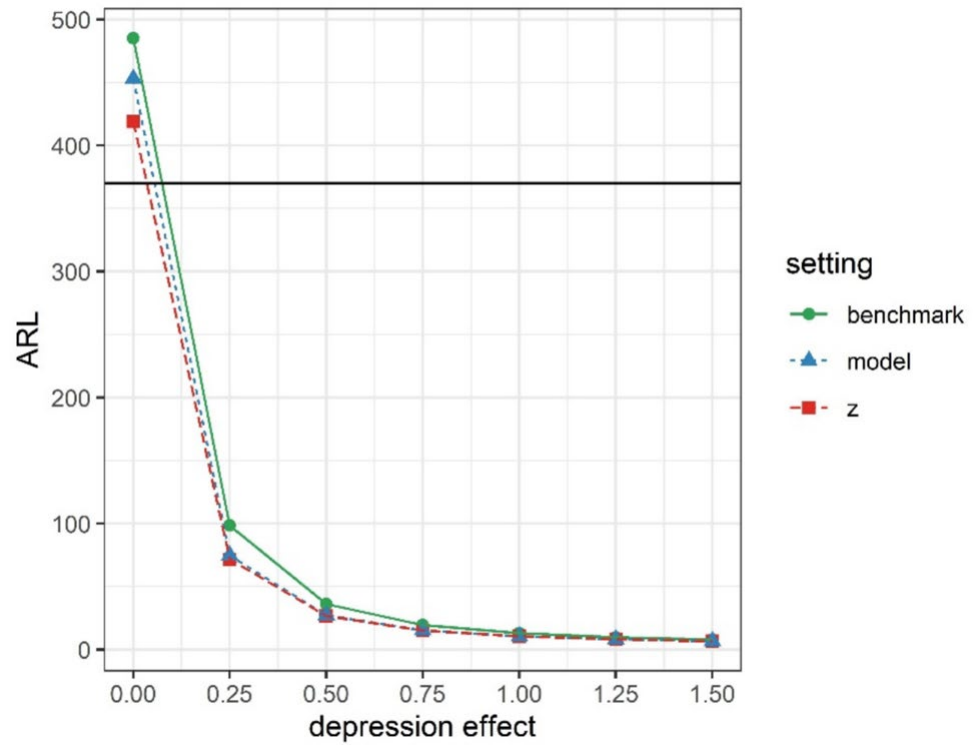
Average run length (ARL) curves for different SPC methods, assuming large contextual effects. The horizontal line denotes the average run length in absence of depression (i.e., the average timing of the first false alarm), which was set at 370

**Fig. 4:**
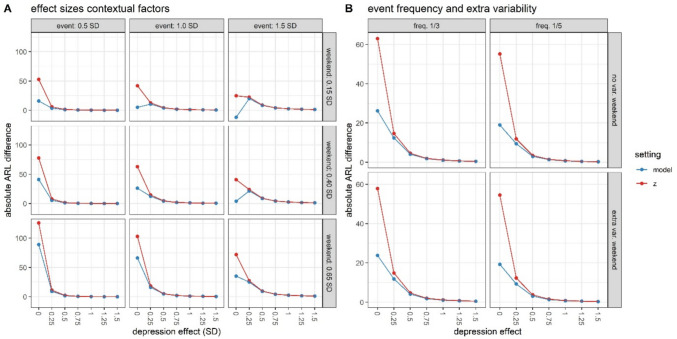
Differences in average run lengths (ARL) for different effect sizes of events and weekends (**A**, assuming an event frequency of 1/3, no additional variability, and no bias in event detection) and event frequency and additional variability during weekends (**B**, assuming moderate event and weekend effects and no bias in event detection). Positive values indicate that the context-sensitive methods detected the change earlier than the standard method, which is advantageous when the depression effect > 0 SD (improved sensitivity) and disadvantageous when the depression effect = 0 SD (worsened specificity)

**Fig. 5:**
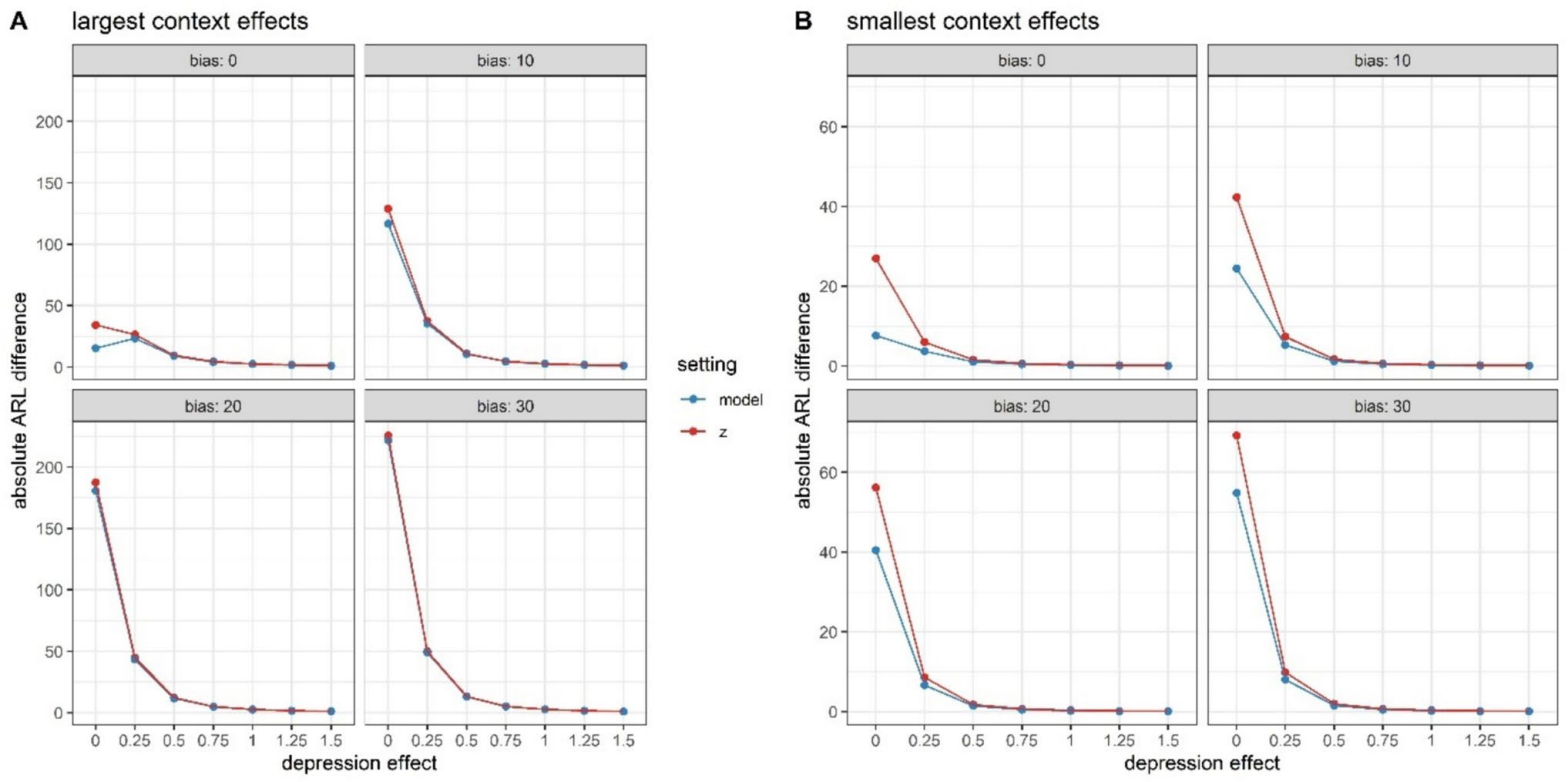
Differences in average run lengths (ARL) for increasing levels of bias, in the case of small and large contextual effects. Positive values indicate that the context-sensitive methods detected the change earlier than the standard method, which is advantageous when the depression effect > 0 SD (improved sensitivity) and disadvantageous when the depression effect = 0 SD (worsened specificity). **A** The smallest context effects were as follows: event effect 0.5 SD, weekend effect 0.15 SD, event frequency 1/5, no additional variability during weekends. With such small effects, context-sensitive methods perform similar to the benchmark method (bias 0). Correspondingly, when introducing bias in the event detection during phase II, the performance of context-sensitive factors is hardly affected. **B** The largest context effects were as follows: event effect 1.5 SD, weekend effect 0.65 SD, event frequency 1/3, additional variability during weekends. In this case, context-sensitive methods outperform the benchmark method (bias 0). However, with increasing bias, ARL values in cases of no depression effect (0 SD) of context-sensitive methods are substantially lower, implying reduced specificity

ARL curves (Fig. 3) show the average timing of the earliest warning sign (ARL) with increasing depression effects. These curves first confirmed that larger effects are easier to detect by means of warning signs: ARL values decline as depression effects increase. Further, on average, context-sensitive methods (model, z) outperformed the benchmark method. Specifically, while the average timing of the first false alarm (depression effect = 0 SD) was comparable across methods (> 370 observations), the average timing of the first true alarm was earlier for context-sensitive methods. Depending on the size of the depression effect, the latter two methods yielded warning signs that were 1–13 (model method) and 16 (z-method) observations earlier than the standard method. This points to increased sensitivity, while maintaining specificity. This comparative advantage of context-sensitive methods came with two nuances. Firstly, the advantage was most pronounced in the presence of strong contextual effects (and especially with strong event effects). This is depicted in Fig. 4A, which shows that the advantage of context-sensitive methods over the standard SPC method declines to zero less quickly for large contextual effects (lower right) than for smaller contextual effects (upper left). Unlike the size of event and weekend effects, event frequency and weekend variability have less impact on the differential performance of context-sensitive methods versus the standard SPC method (Fig. 4B). Secondly, the advantage of context-sensitive methods relied on a lack of bias in the assessment of negative event occurrence in phase II. Specifically, with an increasing proportion of misidentified negative events, false alarms for context-sensitive methods became more likely. This is illustrated in Fig. 5, which shows that context-sensitive methods detect false alarms (i.e., warnings in cases of depression effect = 0) increasingly early as bias increases (see also Fig. S1). Thus, as hypothesized, taking into account contextual information in the implementation of SPC improves the accuracy of warning signs, but only if contextual factors have a substantial effect and are correctly identified.

**Fig. 6:**
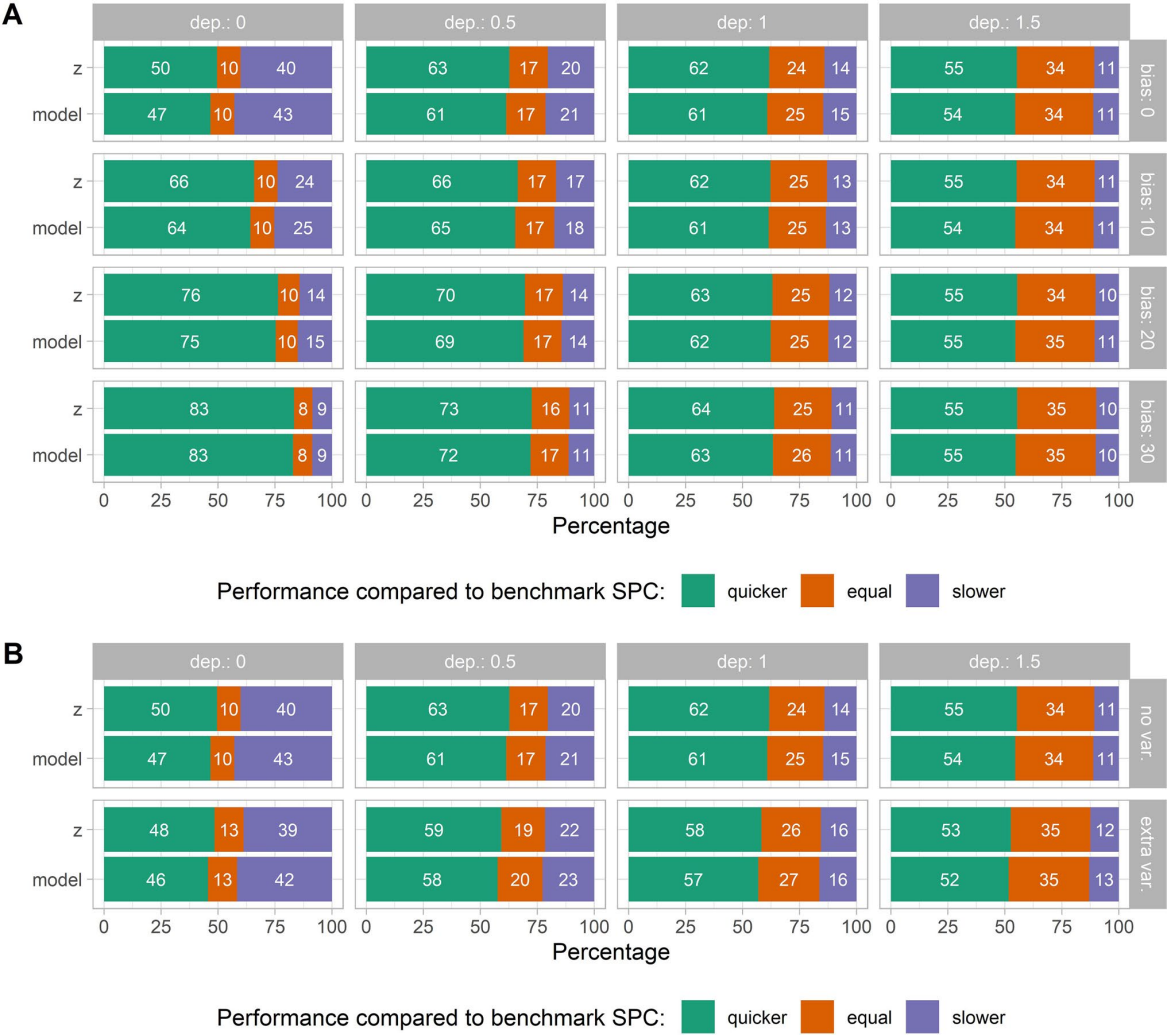
Difference in run length between the context-sensitive methods (z, model) relative to the benchmark method, assuming strong contextual effects. Numbers in the plots denote the percentage of replicates for which each method was quicker than, equally quick as, or slower than the benchmark method. **A** The relative advantage of context-sensitive methods declines as depression effects increase from 0.5 SD (dep.: 0.5) to 1.5 SD (dep.: 1.5). Increasing bias in the detection of events leads to a quicker detection of false alarms (dep.: 0), implying lower specificity. **B** In the case of additional variability during weekends (extra var.), and assuming no bias in event detection, the comparative advantage of context-sensitive methods over the benchmark method hardly changes. A similar figure for other simulated conditions (i.e., smaller and moderate contextual effects) is provided in the supplement, Fig. S2

The advantage of context-sensitive SPC methods relative to the standard method depends not just on the size of contextual effects and the bias in these effects, but also on the size of the depression effect (supplement, Fig. S3). This is illustrated by comparisons of the frequency with which context-sensitive methods are slower than, equally quick as, or quicker than the standard SPC method in detecting imminent depression. In the case of small depression effects (0.5 SD) and strong weekend and event effects^8^, warning signs generated through context-sensitive methods were earlier than those generated by the standard method in 60% (model method) to 62% (z-method) of the replicates, while the context-sensitive methods were equally quick as the benchmark method in around 17–20% of the replicates (Fig. 6). By contrast, in cases of large depression effects (1.5 SD), warning signs generated through context-sensitive methods were less often quicker (namely, in around 52–55% of the replicates) and more often equally quick (35% of the iterations) as compared to the benchmark method. It can be concluded that the advantage of taking into account contextual information gradually declines in the presence of other, stronger effects on momentary emotions (i.e., large depression effects). Within the context-sensitive methods, the z-method appeared superior to the modeling method, as it was more often the quickest to detect impending change (namely, in 8% to 17% of replicates). The relative advantage of the z-method over the modeling method was clearest in the case of small depression effects, and remained similar for different contextual effect sizes. This may be explained by the fact that the linear model defined in the modeling method did not fit the simulated data perfectly, in the sense that contextual effects (${\widehat{\beta }}_{phaseI,1}$ and ${\widehat{\beta }}_{phaseI,2}$) were estimated with some error. To illustrate, small weekend effects (−0.15 SD) were estimated with a standard deviation of 0.128, meaning that in around 12% of the fitted linear models, weekend effects were estimated to be positive (i.e., increased NA) rather than negative. This misestimation of effects in turn led to overestimated residuals, higher control limits, and a lower probability of warning signs. That being said, the average lead of the z-method was small (on average one to three observations for depression effects of 0.25 to 0.5 SD), and the most common scenario was equal performance of both methods (namely, 70% to 87% of the replicates).

### Illustrative empirical case study

**Fig. 7:**
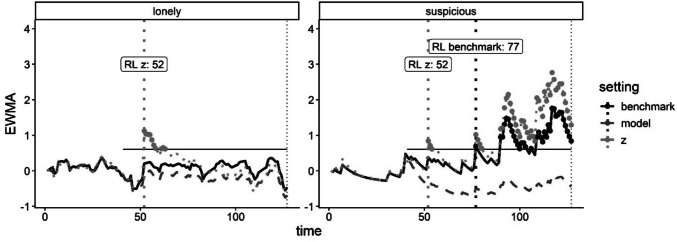
Control charts depicting changes in the monitored score (exponentially moving average, or EWMA) over time for two different mental states in the empirical data. For these mental states, context-sensitive methods outperformed the standard SPC method, which is illustrated by means of vertical lines (denoting the run length, or RL, per method). The vertical dotted line reflects the relapse towards depression. The horizontal line indicates the control limit

In the empirical data, linear regression models showed that during phase I, events had a small to moderate, statistically significant impact on negative mental states (average effect size across mental states = 0.63 SD, range 0.29 – 0.77). By contrast, weekends had more ambiguous effects on negative mental states, and these effects were not statistically significant (average effect size = 0.02 SD, range −0.13 to 0.17; Table S1). The effect of depression onset was small to moderate (average effect size = 0.31 SD, range 0.08–0.67). When applying the different SPC methods (standard, z, model) for 12 different negative mental states, all items yielded warning signs at roughly the same time (Table S1), with two notable exceptions: suspicious (25 days earlier in the z vs. standard method) and lonely (only detected in the z-method; Fig. 7). The reason that the z-method yielded earlier warning signs than both other methods may be that it allows for differing variances across contextual factors. To illustrate, for feeling lonely, the overall variance (used in the standard, or benchmark, SPC method) was equal to 0.27, while the variance on weekdays with events was only 0.07 (vs. 0.29 on weekdays without events; used in the z-method). As a result, high values on event weekdays would remain much higher in the z-method than in the standard method, which translates to warning signs that are easier to detect. While this hints at an advantage of the z-method over the standard method for some mental states, the aforementioned warnings were also short-lived, complicating conclusive inferences.

## Discussion

Applying SPC—which was originally developed to monitor production processes—to monitor repeatedly assessed emotions comes with numerous challenges. This paper addressed the assumption that control limits remain constant over time, even though the boundaries of healthy momentary emotions likely depend on contextual factors (Ong & Leger, 2022). A simulation study comparing the standard SPC method to two newly developed context-sensitive methods revealed that context-sensitive SPC methods outperform the standard method, but only when assuming relatively large contextual effects that can be identified without bias. Reanalysis of openly available empirical data showed that context-sensitive methods often performed equally well as the standard method, with exceptions for which context-sensitive methods yielded earlier warning signs than the standard method. Taken together, the findings of this study tentatively support considering contextual information in future SPC applications.

The improved performance of context-sensitive versus standard methods was not necessarily reflected in a reduced probability of false alarms, but instead in a quicker detection of true alarms. In absolute terms, the improved sensitivity of context-sensitive methods was most pronounced in cases where depression effects were small (i.e., <0.5 SD). In contrast, large depression effects were often detected equally quickly with different SPC methods. It follows that accounting for variance explained by factors other than depression (events, weekends) is particularly relevant in cases where depression itself has a limited impact on momentary emotions. However, it should be noted that the simulated data contained no systematic sources of variance other than those of interest (events, weekends, depression). In reality, there may be many more factors that influence momentary emotions, most of which may remain unobserved. Distinguishing signal (variance explained by depression) from noise (variance explained by all other factors) is therefore critical, meaning that in empirical settings, the advantage of context-sensitive SPC methods over the benchmark SPC method may extend beyond small depression effects.

When zooming in on the performance of context-sensitive SPC methods (z vs. model), we found that both methods very often performed identically. Nonetheless, the z-method may be tentatively favored, given its marginally better performance in simulations and the empirical application. This might be attributable to the ability of the z-method to handle variance differences across contextual factors, which were indeed present in the empirical data. A second advantage of the z-method is that it is likely less dependent on sufficient phase I data than the modeling method, which could be advantageous in empirical settings. That being said, the z-method also comes with the assumption that contextual factors can be categorized (e.g., event: yes/no; weekend: yes/no), which may not always be possible. Another challenge, which pertains to both context-sensitive SPC methods, is that their incremental value may in part depend on how well the standard (benchmark) method performs. In the empirical illustration, for instance, the good performance of the benchmark method may have left little room for improvement, and it would thus be worthwhile to explore the utility of context-sensitive methods in less optimal scenarios. Additionally, context-sensitive methods depend on an accurate measurement of contextual factors, which we expand upon below.

While it seems that context-sensitive SPC methods can in principle be applied without cost, in practice, the utility of context-sensitive SPC methods will depend on our ability to measure contextual information. Specifically, the current work showed that biased contextual factors reduce the specificity of context-sensitive methods, implying increased false alarm rates. Bias in the estimation of contextual factors is particularly relevant for those variables that are hard to quantify objectively, such as negative events. Many ESM studies have assessed such events by retrospectively assessing people’s appraisal (e.g., “Think of the most important event since the last assessment. How pleasant/unpleasant was this event?”) (Li et al., 2024; Vaessen et al., 2019) or by presenting individuals with a checklist (e.g., “Since the last assessment, have you hurried to meet a deadline?”) (Charles et al., 2013; Kanner et al., 1981). An alternative approach is to implement qualitative methods in order to assess negative events (e.g., open text boxes or audio recordings; Almeida, 2005). Further progress in this direction will likely help to gain insight into what individuals experience on a daily basis, and how such experiences influence their momentary emotions. The challenge for SPC-related endeavors will be to explore whether, for a subset of negative events (or individuals), certain “normative reactions” can be defined that then inform adaptions to control limits (or more formally, the monitored scores). While such normative reactions are ubiquitous in clinical reasoning and decision-making (Hardy & Segerstrom, 2017; Klein et al., 2023; Os et al., 2013), they are still difficult to quantify empirically, and thus further research is required to establish exactly when momentary emotions elicited by specific (categories of) negative events may warn of impending mental health problems. An alternative route forward is to focus on contextual factors that may be similarly relevant, but easier to quantify, including medication use (e.g., retrieved from medical records) and passively collected data (e.g., weather, geolocation). Although combining such data with self-reports is not without challenges (Langener et al., 2024), their more straightforward quantification makes them appealing for context-sensitive SPC applications.

A related challenge that was not addressed currently, but might be relevant for further advancing context-sensitive SPC methods, is that events may have a sustained impact on negative affect. This was not considered in the present work, as we simulated one assessment per day and only took into account the effect of negative events on *current* emotions (i.e., events that happened today on emotions registered today). The resulting peaks (due to negative events) were likely smoothed due to the EWMA method, which in turn may have reduced the impact of negative events and thereby reduced the comparative advantage of context-sensitive methods. Follow-up research is needed to evaluate context-sensitive SPC methods in the presence of sustained effects of contextual factors, which is not trivial since it would require assumptions regarding how long contextual factors influence negative affect. A more straightforward route may therefore be to explore the influence of contextual factors on lagged associations between emotions (Bringmann et al., 2024).

## Limitations and considerations

In order to examine the comparative performance of context-sensitive (vs. standard) SPC methods, we imposed several simplifications. The most prominent concerned limiting contextual factors to events and weekends and modeling events as binary variables. The latter was required for the z-method, which unlike the modeling method can only handle categorical contextual variables. Regarding the former point, many other contextual factors could also be relevant to momentary emotions, including environmental factors (e.g., weather, societal developments, geolocation), social factors (e.g., company), and biological rhythms (e.g., menstrual cycle, cortisol cycle). While such alternative factors might have been interesting from a more applied point of view, including them in the current simulation design would have required many (additional) assumptions, without a proportionate impact on the potential inferences or conclusions. This links to a second limitation, namely the adoption of assumptions that may complicate generalizability to empirical settings. These include, but are not limited to, the assumption that depression may occur in a stepwise (rather than gradual) fashion (Smit, Snippe et al., 2023b), the assumption that the frequency and intensity of events does not change with changing depression status, and the lack of temporal dependencies in the simulated data (e.g., prolonged effects of events on momentary emotions). Further, the simulation did not consider the presence of missing observations, which could be contingent on contextual factors. For instance, individuals may miss more assessments during weekends than on weekdays or miss assessments in noisy surroundings (e.g., when in company) (Reiter & Schoedel, 2023). A final simplification that warrants attention is that—like other SPC studies within and outside of clinical psychology—we evaluated SPC by its ability to generate true alarms as early as possible and false alarms as late as possible, regardless of whether those alarms (warning signs) were sustained. Yet, it can be questioned whether single, isolated warnings that do not persist over time should be trusted, highlighting the need for follow-up empirical research into *when* and *for how long* warning signs should emerge in order to be considered “warnings.” Relatedly, it should be acknowledged that—depending on the outcome that is being anticipated—alternative performance criteria (e.g., favoring high specificity over sensitivity) might be preferred (Ludwig et al., 2025). In conclusion, the current work should be viewed primarily as a methodological illustration of how SPC may be tailored to ESM data, which may hold promise for real-time monitoring in depression research and beyond (e.g., suicidality, binge eating, substance use disorders). Follow-up research is necessary to address the open questions that remain.

## Conclusions

SPC enables real-time monitoring of ESM data, but requires attunement to acknowledge that healthy emotional experiences depend on context. The current work shows that incorporating contextual information into SPC may indeed improve the detection of warning signs for impending depression. In practice, the added value of context-sensitive SPC methods will depend on how well context can be measured empirically. This measurement still leaves room for improvement, and the current work therefore encourages further research into determining context-appropriate emotions. In parallel, more research is needed to improve our understanding of *when* SPC-derived warning signs are actually a “warning” (e.g., when do they first emerge, [how long] should they persist). The resulting findings may help to innovate SPC methods and, in the longer run, improve the real-time detection of worsening depression.

## Supplementary Information

Below is the link to the electronic supplementary material.

Supplementary file1 (DOCX 1203 KB)

## Data Availability

Simulated data is available on the Open Science Framework: https://osf.io/3n4tu. Empirical data is available online (Kossakowski et al., 2017: Data from “Critical slowing down as a personalized warning sign for depression”, Open Psychology Data).

## Footnotes

1 Of note, we will selectively focus on exceeding the upper (not the lower) control limit, and thus apply a one-sided procedure. This follows from the logic that, when monitoring negative emotions prior to imminent depression, warnings should be informed by “too high” but not “too low” levels (Schat et al., 2023; Schreuder et al., 2024; Smit & Snippe, 2023; Snippe et al., 2023).

2 We here assume that “the earlier, the better,” but this assumption can be questioned. In practice, the desired timing of warning signs will depend on the ensuing intervention. Additionally, it should be noted that SPC formally aims to detect rising *risk* of depression. We shortened this “detecting depression” in the interest of readability.

3 Note that, with $\lambda$=1, historical ratings are not at all taken into account, meaning that the EWMA score fully depends on the current rating. In this case, the EWMA SPC method is identical to the Shewhart SPC method, which monitors raw ratings rather than weighted averages (Montgomery, 2009; Schat et al., 2023; Shewhart, 1931).

4 Note that ARL curves capture both the sensitivity and specificity of warning signs. Whether both are equally important, however, depends on the outcome that is being anticipated and the costs of missed versus false warning signs. Specifically, while high sensitivity ensures that an outcome of interest is always anticipated by warning signs (which is important when missed alarms are costly, e.g. in the case of predicting suicidality), high specificity ensures the absence of costly false alarms (which is important for retaining trust in monitoring, and has been considered a priority in earlier work; Ludwig et al., 2025).

5 To compare effects across studies, we divided effects by the average within-person standard deviation reported in the study. In cases where the latter was not reported, we assumed an average within-person standard deviation of 10% of the original scale, which is in line with the current simulation setup (scale 0–100, within-person SD of 10) and with empirical findings (Dejonckheere et al., 2019). For studies that assessed negative events through ordinal or continuous scales, we assumed that the midpoint of the scale (e.g., on a scale from 0 to 100, 50 points) reflected the difference between 0 and 1.

6 A study that assessed both self-reported and observer-reported stressors reported that the correlation between the two was only moderate (*r* = 0.36) (Almeida et al., 2002).

7 We also inspected the influence of heightened variability during weekdays (as opposed to weekend days). This yielded results with a similar pattern to those pertaining to the addition of weekend variability, and we therefore report the latter in the main text, and included the results for additional variability during weekdays in the supplement.

8 We here assumed additional weekend variability, no bias in the detection of events, and an event frequency of 1/3. A table with full results for other simulated variations is provided in supplemental material (Table S2).

## Code availability

Code for analysis is available on the Open Science Framework: https://osf.io/3n4tu.

## References

[CR1] Almeida, D. M. (2005). Resilience and vulnerability to daily stressors assessed via diary methods. *Current Directions in Psychological Science,**14*(2), 64–68. 10.1111/j.0963-7214.2005.00336.x

[CR2] Almeida, D. M., Wethington, E., & Kessler, R. C. (2002). The daily inventory of stressful events: An interview-based approach for measuring daily stressors. *Assessment,**9*(1), 41–55. 10.1177/1073191102091006 11911234 10.1177/1073191102091006

[CR3] Anderson, S. F., Sladek, M. R., & Doane, L. D. (2021). Negative affect reactivity to stress and internalizing symptoms over the transition to college for Latinx adolescents: Buffering role of family support. *Development and Psychopathology,**33*(4), 1322–1337. 10.1017/S095457942000053X 32611477 10.1017/S095457942000053X

[CR4] Areni, C. S., Burger, M., & Zlatevska, N. (2011). Factors affecting the extent of monday blues: Evidence from a meta-analysis. *Psychological Reports,**109*(3), 723–733. 10.2466/13.20.PR0.109.6.723-733 22420107 10.2466/13.20.PR0.109.6.723-733

[CR5] Bai, S., Robles, T. F., Reynolds, B. M., & Repetti, R. L. (2020). Daily mood reactivity to stress during childhood predicts internalizing problems three years later. *Journal of Abnormal Child Psychology,**48*(8), 1063–1075. 10.1007/s10802-020-00650-7 32328865 10.1007/s10802-020-00650-7

[CR6] Ben-Zeev, D., Young, M. A., & Madsen, J. W. (2009). Retrospective recall of affect in clinically depressed individuals and controls. *Cognition and Emotion,**23*(5), 1021–1040. 10.1080/02699930802607937

[CR7] Booij, S. H., Snippe, E., Jeronimus, B. F., Wichers, M., & Wigman, J. T. W. (2018). Affective reactivity to daily life stress: Relationship to positive psychotic and depressive symptoms in a general population sample. *Journal of Affective Disorders,**225*, 474–481. 10.1016/j.jad.2017.08.051 28863300 10.1016/j.jad.2017.08.051

[CR8] Bos, F. M., Schreuder, M. J., George, S. V., Doornbos, B., Bruggeman, R., van der Krieke, L., . . . Snippe, E. (2022). Anticipating manic and depressive transitions in patients with bipolar disorder using early warning signals. *International Journal of Bipolar Disorders,**10*(1), Article 12. 10.1186/s40345-022-00258-4 35397076 10.1186/s40345-022-00258-4 PMC8994809

[CR9] Bouwmans, M. E. J., Bos, E. H., Hoenders, H. J. R., Oldehinkel, A. J., & de Jonge, P. (2017). Sleep quality predicts positive and negative affect but not vice versa. An electronic diary study in depressed and healthy individuals. *Journal of Affective Disorders,**207*, 260–267. 10.1016/j.jad.2016.09.046 27736737 10.1016/j.jad.2016.09.046

[CR10] Box, G., & Paniagua-Quinones, C. (2007). Two charts: Not one. *Quality Engineering,**19*(2), 93–100. 10.1080/08982110701241590

[CR11] Bringmann, L. F., Ariens, S., Ernst, A. F., Snippe, E., & Ceulemans, E. (2024). Changing networks: Moderated idiographic psychological networks. *Advances.in/Psychology,**2*, e658296. 10.56296/aip00014

[CR12] Bylsma, L. M., Taylor-Clift, A., & Rottenberg, J. (2011). Emotional reactivity to daily events in major and minor depression. *Journal of Abnormal Psychology,**120*(1), 155–167. 10.1037/a0021662 21319928 10.1037/a0021662

[CR13] Charles, S. T., Piazza, J. R., Mogle, J., Sliwinski, M. J., & Almeida, D. M. (2013). The wear-and-tear of daily stressors on mental health. *Psychological Science,**24*(5), 733–741. 10.1177/0956797612462222 23531486 10.1177/0956797612462222 PMC3654031

[CR14] De Calheiros Velozo, J., Lafit, G., Viechtbauer, W., van Amelsvoort, T., Schruers, K., Marcelis, M., . . . Vaessen, T. (2023). Delayed affective recovery to daily-life stressors signals a risk for depression. *Journal of Affective Disorders,**320*, 499–506. 10.1016/j.jad.2022.09.136 36208689 10.1016/j.jad.2022.09.136

[CR15] de Ketelaere, B., Mertens, K., Mathijs, F., Diaz, D. S., & de Baermaeker, J. (2011). Nonstationarity in statistical process control—Issues, cases, ideas. *Applied Stochastic Models in Business and Industry,**27*(4), 367–376. 10.1002/asmb.911

[CR16] Dejonckheere, E., Mestdagh, M., & Houben, M., et al. (2019). Complex affect dynamics add limited information to the prediction of psychological well-being. *Nature Human Behaviour, 3*(5):478–491. 10.1038/s41562-019-0555-0 10.1038/s41562-019-0555-0 30988484

[CR17] Díaz-Morales, J. F., & Parra-Robledo, Z. (2021). Day-of-week mood patterns in adolescents considering chronotype, sleep length and sex. *Personality and Individual Differences,**179*, 110951. 10.1016/j.paid.2021.110951

[CR18] Drake, A., Dore, B. P., Falk, E. B., Zurn, P., Bassett, D. S., & Lydon-Staley, D. M. (2022). Daily stressor-related negative mood and its associations with flourishing and daily curiosity. *Journal of Happiness Studies,**23*(2), 423–438. 10.1007/s10902-021-00404-2 40641736 10.1007/s10902-021-00404-2 PMC12245169

[CR19] Elmer, T., Wolf, M., Snippe, E., & Scholz, U. (2025). A social support just-in-time adaptive intervention for individuals with elevated depressive symptoms: A feasibility study with micro-randomized trial design. 10.2196/74103 10.2196/74103 PMC12421209 40857095

[CR20] Gunthert, K. C., Cohen, L. H., Butler, A. C., & Beck, J. S. (2005). Predictive role of daily coping and affective reactivity in cognitive therapy outcome: Application of a daily process design to psychotherapy research. *Behavior Therapy,**36*(1), 77–88. 10.1016/S0005-7894(05)80056-5

[CR21] Hardy, J., & Segerstrom, S. C. (2017). Intra-individual variability and psychological flexibility: Affect and health in a National US sample. *Journal of Research in Personality*. 10.1016/j.jrp.2016.04.002

[CR22] Harvey, B., Milyavskaya, M., Hope, N., Powers, T. A., Saffran, M., & Koestner, R. (2015). Affect variation across days of the week: Influences of perfectionism and academic motivation. *Motivation and Emotion,**39*(4), 521–530. 10.1007/s11031-015-9480-3

[CR23] Helliwell, J. F., & Wang, S. (2015). How was the weekend? How the Social Context Underlies Weekend Effects in Happiness and Other Emotions for US Workers. *PLoS One,**10*(12), e0145123. 10.1371/journal.pone.0145123 26699709 10.1371/journal.pone.0145123 PMC4689414

[CR24] Helmich, M. A., Smit, A. C., Bringmann, L. F., Schreuder, M. J., Oldehinkel, A. J., Wichers, M., & Snippe, E. (2023). Detecting impending symptom transitions using early warning signals in individuals receiving treatment for depression. *Clinical Psychological Science,**11*(6), 994–1010. 10.1177/21677026221137006

[CR25] Hülsheger, U. R., Uitdewilligen, S., Zijlstra, F. R. H., & Walkowiak, A. (2022). Blue monday, yellow friday? Investigating work anticipation as an explanatory mechanism and boundary conditions of weekly affect trajectories. *Journal of Occupational Health Psychology,**27*(4), 359–376. 10.1037/ocp0000330. (2022-62543-001). 35588381 10.1037/ocp0000330

[CR26] Kanner, A. D., Coyne, J. C., Schaefer, C., & Lazarus, R. S. (1981). Comparison of two modes of stress measurement: Daily hassles and uplifts versus major life events. *Journal of Behavioral Medicine,**4*(1), 1–39. 10.1007/BF00844845 7288876 10.1007/BF00844845

[CR27] Klein, R. J., Jacobson, N. C., & Robinson, M. D. (2023). A psychological flexibility perspective on well-being: Emotional reactivity, adaptive choices, and daily experiences. *Emotion,**23*(4), 911–924. 10.1037/emo0001159 36048033 10.1037/emo0001159 PMC10035040

[CR28] Knoth, S. (2020). *spc: Statistical process control - calculation of ARL and other control chart performance measures (0.6.4)*. https://cran.r-project.org/package=spc

[CR29] Kossakowski, J. J., Groot, P. C., Haslbeck, J. M. B., Borsboom, D., & Wichers, M. (2017). Data from ‘Critical slowing down as a personalized early warning signal for depression.’ *Journal of Open Psychology Data*, *5*. 10.5334/jopd.29

[CR30] Kraiss, J. T., Vaessen, T., & Klooster, P. M. ten. (2024). Idiographic bidirectional associations of stressfulness of events and negative affect in daily life as indicators for mental health: An experience sampling study. *Stress and Health*, 1–12. 10.1002/smi.3433 10.1002/smi.3433 38817035

[CR31] Lamers, F., Swendsen, J., Cui, L., Husky, M., Johns, J., Zipunnikov, V., & Merikangas, K. R. (2018). Mood reactivity and affective dynamics in mood and anxiety disorders. *Journal of Abnormal Psychology,**127*(7), 659–669. 10.1037/abn0000378 30335438 10.1037/abn0000378 PMC12837089

[CR32] Langener, A. M., Stulp, G., Jacobson, N. C., Costanzo, A., Jagesar, R. R., Kas, M. J., & Bringmann, L. F. (2024). It’s all about timing: Exploring Different Temporal Resolutions for Analyzing Digital-Phenotyping Data. *Advances in Methods and Practices in Psychological Science,**7*(1), 25152459231202676. 10.1177/25152459231202677

[CR33] Li, X., Vaessen, T., Lafit, G., Van Aubel, E., Hiekkaranta, A. P., Houben, M., Beijer-Klippel, A., De Haan, L., Schirmbeck, F., Reininghaus, U., & Myin-Germeys, I. (2024). Higher emotion regulation flexibility predicts more stable negative emotions and faster affective recovery in early psychosis: An experience sampling study. *Psychological Medicine*. 10.1017/S0033291724000151 38343379 10.1017/S0033291724000151

[CR34] Ludwig, V. M., Bittendorf, C. A., Reinhard, I., Guth, M., Mühlbauer, E., Severus, . . . Ebner-Priemer, U. W. (2025). Predicting depressive and manic episodes in patients with bipolar disorder using statistical process control methods on passive sensing data. *Journal of Psychopathology and Clinical Science,**134*(8), 971–981. 10.1037/abn0001002 40689911 10.1037/abn0001002

[CR35] Machell, K. A., Kashdan, T. B., Short, J. L., & Nezlek, J. B. (2015). Relationships between meaning in life, social and achievement events, and positive and negative affect in daily life. *Journal of Personality,**83*(3), 287–298. 10.1111/jopy.12103 24749860 10.1111/jopy.12103

[CR36] Montgomery, D. C. (2009). *Introduction to statistical quality control* (6th ed.). John Wiley & Sons.

[CR37] Myin-Germeys, I., Kasanova, Z., Vaessen, T., Vachon, H., Kirtley, O., Viechtbauer, W., & Reininghaus, U. (2018). Experience sampling methodology in mental health research: New insights and technical developments. *World Psychiatry,**17*, 123–132. 29856567 10.1002/wps.20513 PMC5980621

[CR38] Nelson, J., Klumparendt, A., Doebler, P., & Ehring, T. (2018). Everyday emotional dynamics in major depression. *Emotion,**20*, 179–191. 10.1037/emo0000541 30589297 10.1037/emo0000541

[CR39] Ong, A. D., & Leger, K. A. (2022). Advancing the study of resilience to daily stressors. *Perspectives on Psychological Science,**17*(6), 1591–1603. 10.1177/17456916211071092 35748196 10.1177/17456916211071092 PMC10122438

[CR40] Os, Jvan, Delespaul, P., Wigman, J. T. W., Myin-Germeys, I., & Wichers, M. (2013). Psychiatry beyond labels: Introducing contextual precision diagnosis across stages of psychopathology. *Psychological Medicine,**43*, 1563–1567. 10.1017/s0033291713000937 23769385 10.1017/S0033291713000937

[CR41] Peeters, F., Nicolson, N. A., Berkhof, J., Delespaul, P., & De Vries, M. (2003). Effects of daily events on mood states in major depressive disorder. *Journal of Abnormal Psychology,**112*(2), 203–211. 10.1037/0021-843X.112.2.203 12784829 10.1037/0021-843x.112.2.203

[CR42] Reiter, T., & Schoedel, R. (2023). Never miss a beep: Using mobile sensing to investigate (non-)compliance in experience sampling studies. *Behavior Research Methods,**56*(4), 4038–4060. 10.3758/s13428-023-02252-9 37932624 10.3758/s13428-023-02252-9 PMC11133120

[CR43] Ryan, R. M., Bernstein, J. H., & Brown, K. W. (2010). Weekends, work, and well-being: Psychological need satisfactions and day of the week effects on mood, vitality, and physical symptoms. *Journal of Social and Clinical Psychology,**29*(1), 95–122. 10.1521/jscp.2010.29.1.95

[CR44] Schat, E., Tuerlinckx, F., Smit, A. C., De Ketelaere, B., & Ceulemans, E. (2023). Detecting mean changes in experience sampling data in real-time: A comparison of univariate and multivariate statistical process control methods. *Psychological Methods,**28*(6), 1335–1357. 10.1037/met0000447 34914467 10.1037/met0000447

[CR45] Schat, E., Tuerlinckx, F., De Ketelaere, B., & Ceulemans, E. (2024). Real-time detection of mean and variance changes in experience sampling data: A comparison of existing and novel statistical process control approaches. *Behavior Research Methods,**56*, 1459–1475. 10.3758/s13428-023-02103-7 37118646 10.3758/s13428-023-02103-7

[CR46] Schat, E., Tuerlinckx, F., Schreuder, M. J., De Ketelaere, B., & Ceulemans, E. (2024). Forecasting the onset of depression with limited baseline data only: A comparison of a person-specific and a multilevel modeling based EWMA approach. *Psychological Assessment,**36*, 379–394. 10.1037/pas0001314 38829348 10.1037/pas0001314

[CR47] Schreuder, M. J., Hartman, C. A., Groen, R. N., Smit, A. C., Wichers, M., & Wigman, J. T. W. (2023). Anticipating transitions in mental health in at-risk youths: A 6-month daily diary study into early-warning signals. *Clinical Psychological Science*, *11*(6). 10.1177/21677026221103138

[CR48] Schreuder, M., Schat, E., Smit, Arnout, Snippe, E., & Ceulemans, E. (2024). Monitoring emotional intensity and variability to forecast depression recurrence in real time in remitted adults. *Journal of Consulting and Clinical Psychology,**92*(8), 505–516. 10.1037/ccp0000871 38512172 10.1037/ccp0000871

[CR49] Schricker, I. F., Nayman, S., Reinhard, I., & Kuehner, C. (2023). Reactivity toward daily events: Intraindividual variability and change in recurrent depression – A measurement burst study. *Behaviour Research and Therapy*, *168*. 10.1016/j.brat.2023.104383 10.1016/j.brat.2023.104383 37586185

[CR50] Sheets, E. S., & Armey, M. F. (2020). Daily interpersonal and noninterpersonal stress reactivity in current and remitted depression. *Cognitive Therapy and Research,**44*(4), 774–787. 10.1007/s10608-020-10096-2

[CR51] Shewhart, W. A. (1931). *Economic control of quality of manufactured product*. Braunworth & Co.

[CR52] Smit, A. C., & Snippe, E. (2023). Real-time monitoring of increases in restlessness to assess idiographic risk of recurrence of depressive symptoms. *Psychological Medicine,**53*, 5060–5069. 10.1017/S0033291722002069 35833374 10.1017/S0033291722002069 PMC10476069

[CR53] Smit, A. C., Schat, E., & Ceulemans, E. (2023). The exponentially weighted moving average procedure for detecting changes in intensive longitudinal data in psychological research in real-time: A tutorial showcasing potential applications. *Assessment,**30*(5), 1354–1368. 10.1177/10731911221086985 35603660 10.1177/10731911221086985 PMC10248291

[CR54] Smit, A. C., Snippe, E., Bringmann, L. F., Hoenders, H. J. R., & Wichers, M. (2023). Transitions in depression: If, how, and when depressive symptoms return during and after discontinuing antidepressants. *Quality of Life Research,**32*, 1295–1306. 10.1007/s11136-022-03301-0 36418524 10.1007/s11136-022-03301-0 PMC10123048

[CR55] Snippe, E., Smit, A., Kuppens, P., Burger, H., & Ceulemans, E. (2023). Recurrence of depression can be foreseen by monitoring mental states with statistical process control. *Journal of Psychopathology and Clinical Science*, *132*(2). 10.1037/abn0000812 10.1037/abn0000812 36808958

[CR56] Stone, A. A., Schneider, S., & Harter, J. K. (2012). Day-of-week mood patterns in the United States: On the existence of ‘Blue Monday’, ‘Thank God it’s Friday’ and weekend effects. *The Journal of Positive Psychology,**7*(4), 306–314. 10.1080/17439760.2012.691980

[CR57] Thompson, R. J., Mata, J., Jaeggi, S. M., Buschkuehl, M., Jonides, J., & Gotlib, I. H. (2012). The everyday emotional experience of adults with major depressive disorder: Examining emotional instability, inertia, and reactivity. *Journal of Abnormal Psychology,**29*(4), 997–1003. 10.1016/j.biotechadv.2011.08.021 10.1037/a0027978 PMC3624976 22708886

[CR58] Vaessen, T., Viechtbauer, W., van der Steen, Y., Gayer-Anderson, C., Kempton, M. J., Valmaggia, L., . . . Myin-Germeys, I. (2019). Recovery from daily-life stressors in early and chronic psychosis. *Schizophrenia Research*, *213*, 32–39. 10.1016/j.schres.2019.03.011 10.1016/j.schres.2019.03.011 30930036

[CR59] Van Der Stouwe, E. C. D., Groenewold, N. A., Bos, E. H., De Jonge, P., Wichers, M., & Booij, S. H. (2019). How to assess negative affective reactivity to daily life stress in depressed and nondepressed individuals? *Psychiatry Research,**279*, 259–266. 10.1016/j.psychres.2019.03.040 31003712 10.1016/j.psychres.2019.03.040

[CR60] Von Klipstein, L., Servaas, M. N., Lamers, F., Schoevers, R. A., Wardenaar, K. J., & Riese, H. (2023). Increased affective reactivity among depressed individuals can be explained by floor effects: An experience sampling study. *Journal of Affective Disorders,**334*, 370–381. 37150221 10.1016/j.jad.2023.04.118

[CR61] Wichers, M., Groot, P. C., Psychosystems ESM group, & EWS group. (2016). Critical slowing down as a personalized early warning signal for depression. *Psychotherapy and Psychosomatics,**85*, 114–116. 10.1159/000441458 26821231 10.1159/000441458

[CR62] Zhaoyang, R., Scott, S. B., Smyth, J. M., Kang, J.-E., & Sliwinski, M. J. (2020). Emotional responses to stressors in everyday life predict long-term trajectories of depressive symptoms. *Annals of Behavioral Medicine,**54*(6), 402–412. 10.1093/abm/kaz057 31794010 10.1093/abm/kaz057 PMC7246260

