# Supplementary material for: Detecting warning signs for psychopathology in real time while accounting for context: Two novel statistical process control applications
Source: Behav Res Methods. 2026 Apr 21;58(5):132. doi: 10.3758/s13428-026-03004-1 (PMC13099871; doi:10.3758/s13428-026-03004-1)
Supplement: Supplementary file 1 — Supplementary file1 (DOCX 1203 KB) [file 13428_2026_3004_MOESM1_ESM.docx]

# Supplement: Detecting warning signs for psychopathology in real-time while accounting for context: two novel statistical process control applications

## M.J. Schreuder, E. Schat, E. Ceulemans

### S1. Rationale for contextual factors

The current study illustrates how accounting for contextual factors – namely, negative events and days of the week – may improve the performance of statistical process control (SPC). The methods we describe are not limited to these contextual factors, but instead could be applied to account for any time-varying variable (e.g., changes in medication). Below, we provide a brief overview of earlier literature that investigated how the currently considered contextual factors may impact momentary emotions – and thus, should be accounted for when applying SPC.

Emotions in response to negative events

There exist different views on what constitutes an appropriate emotional response to negative events. First, it has been argued that mental health is characterized by emotional flexibility (Klein et al., 2023). By contrast, depression may be featured by emotional rigidity (Servaas et al., 2021) or context insensitivity (Kashdan & Rottenberg, 2010), meaning that individuals may get stuck in certain (negative) emotions and have difficulty in adjusting their emotions according to changing contexts. Correspondingly, depressed individuals perceive themselves as less emotionally reactive (Köhling et al., 2016), which has also consistently been found in lab studies (Bylsma et al., 2008). Experience sampling research, however, showed that depression is characterized by heightened instead of blunted emotional reactivity to negative events, which could be indicative of increased stress sensitivity (Booij et al., 2018; Charles et al., 2013; Gunthert et al., 2005; Kraiss et al., 2024; Lamers et al., 2018; Sheets & Armey, 2020; Van Der Stouwe et al., 2019). In agreement, physiological studies showed heightened HPA-axis activity in depressed individuals compared to healthy controls (Pariante & Lightman, 2008). Taken together, it might be that both hypo- and hyperreactivity to negative events are maladaptive, implying that mental health features moderate responses to negative events (Rush et al., 2024).

Emotions in response to temporal cycles

Earlier studies that examined day-of-week effects showed that, in line with popular notions, people tend to feel better on (anticipated) weekend days compared to weekdays (Tsai, 2019). Such “blue Monday” and “thank God it’s Friday” effects are especially clear in non-retired individuals with demanding jobs (Helliwell & Wang, 2015; Hülsheger et al., 2022; Stone et al., 2012). Other factors that influence the effect of weekdays on emotions include individuals’ personality and motivation (Harvey et al., 2015; Larsen & Kasimatis, 1990), need satisfaction (Ryan et al., 2010), and student status (Areni et al., 2011). Although day-of-week effects are generally small in size (Areni et al., 2011; Haqiqatkhah & Hamaker, 2024; Harvey et al., 2015; Pierson et al., 2021), failing to account for weekdays may lead us to mistake naturally occurring emotional fluctuations for warning signs (or vice versa, to mistake warning signs for normal emotional fluctuations). It is therefore worthwhile to investigate whether the detection of warning signs for recurrent depression improves when taking into account day-of-week effects.

**
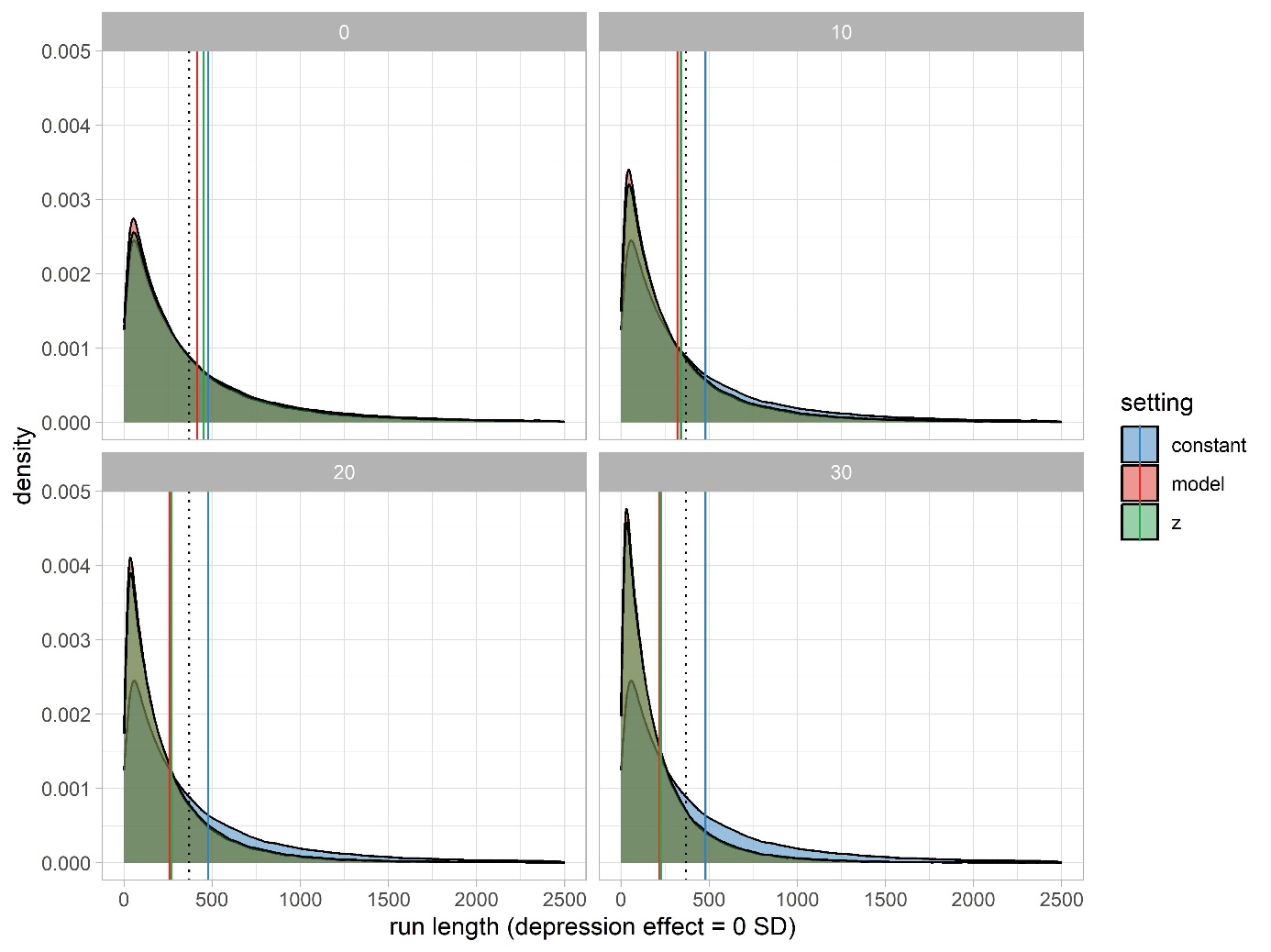
**

*Fig. S1. Run lengths in case of stable remission (depression effect 0 SD) across different levels of bias in the detection of events, assuming no weekend variability, an event frequency of 1/3, and moderate contextual effects.* The benchmark SPC method is not affected by bias in event detection, as it does not take into account events (average run length remains 479). The context-sensitive methods are affected by bias, with an average run length that drops from 416 (z) and 452 (model) to 217 (z) and 225 (model). The black dotted line denotes the expected run length in case of stable remission, namely 370.

*
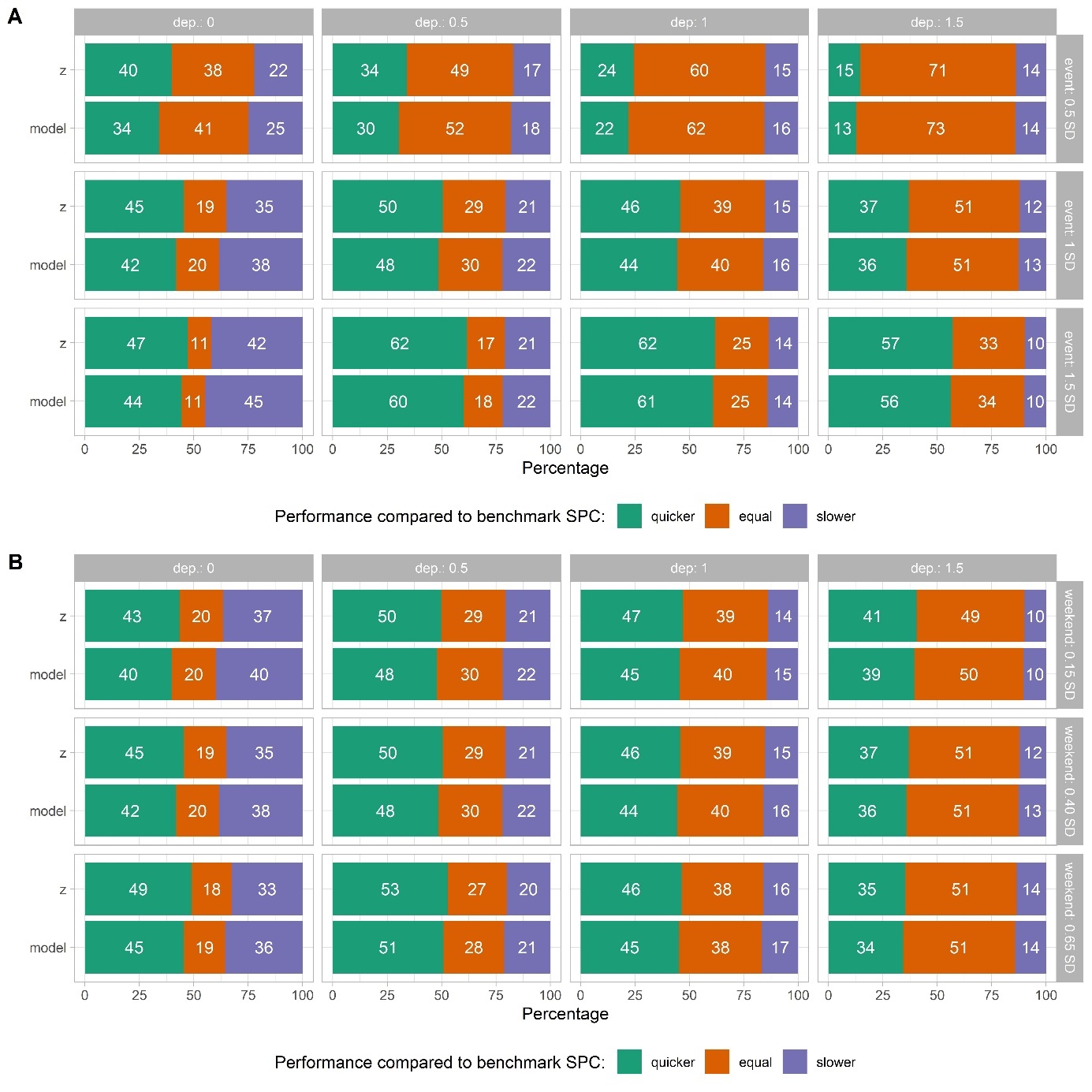
*

*Fig. S2. Differences in run length between the context-sensitive methods (z, model) relative to the benchmark method, assuming no bias in the detection of events, an event frequency of 1/3, and no extra weekend variability.* Numbers in the plots denote the percentage of iterations for which each context-sensitive method was quicker than, equally quick as, or slower than the benchmark method.


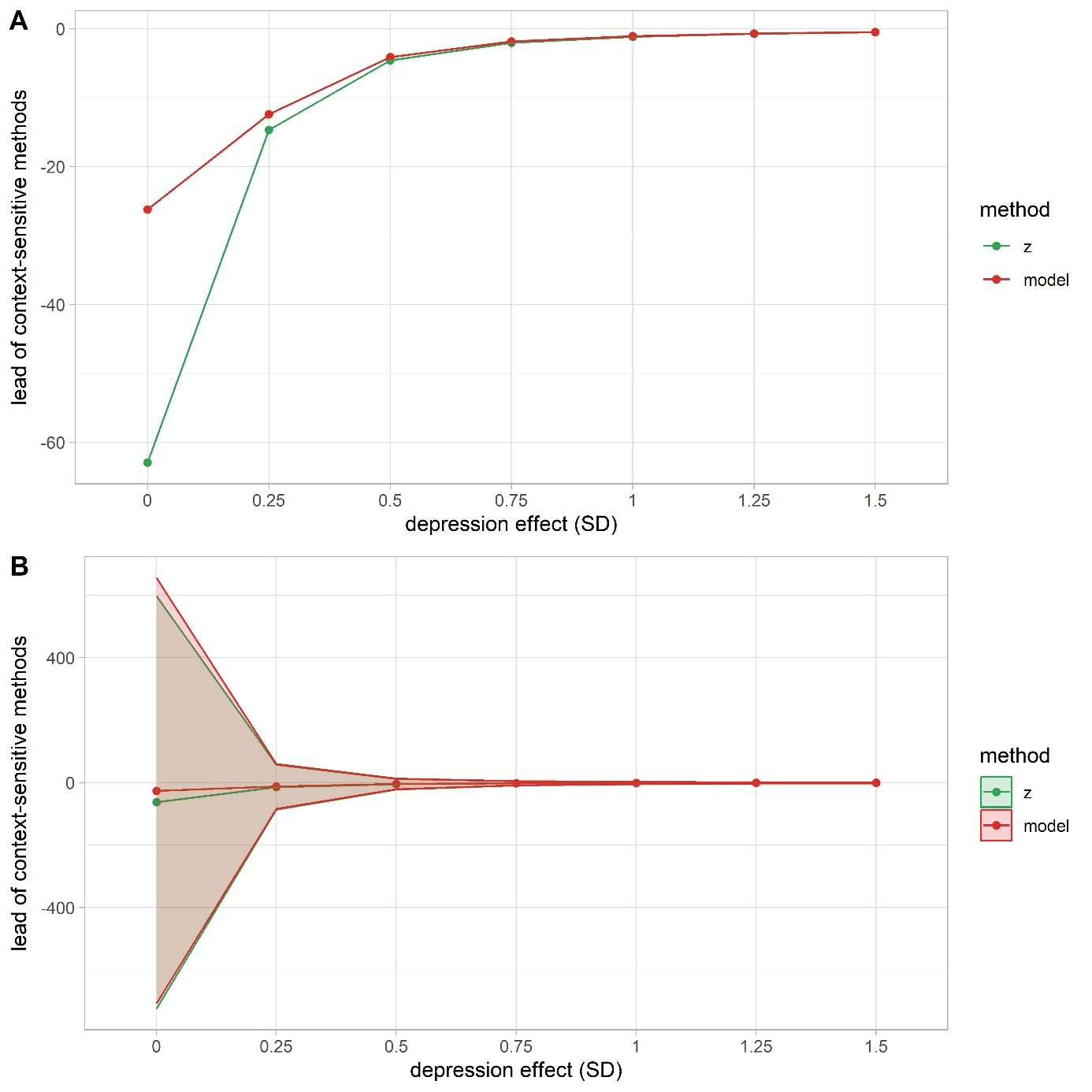
*Fig. S3. Average difference in run length of context-sensitive methods relative to the benchmark method, assuming no bias in the detection of events, no weekend variability, an event frequency of 1/3, and moderate contextual effects.* On average, the context-sensitive methods are quicker than the benchmark method, and this is especially pronounced for smaller depression effects. A) The z-method detects small depression effects (2.5-5 SD) quicker than the model method, but also yields a false alarm (depression effect 0 SD) more quickly. B) This shows the standard deviation around average run length differences, illustrating that the average difference between the z- and model methods is not statistically significant. Table S2 shows the average run length differences for all simulated variations.


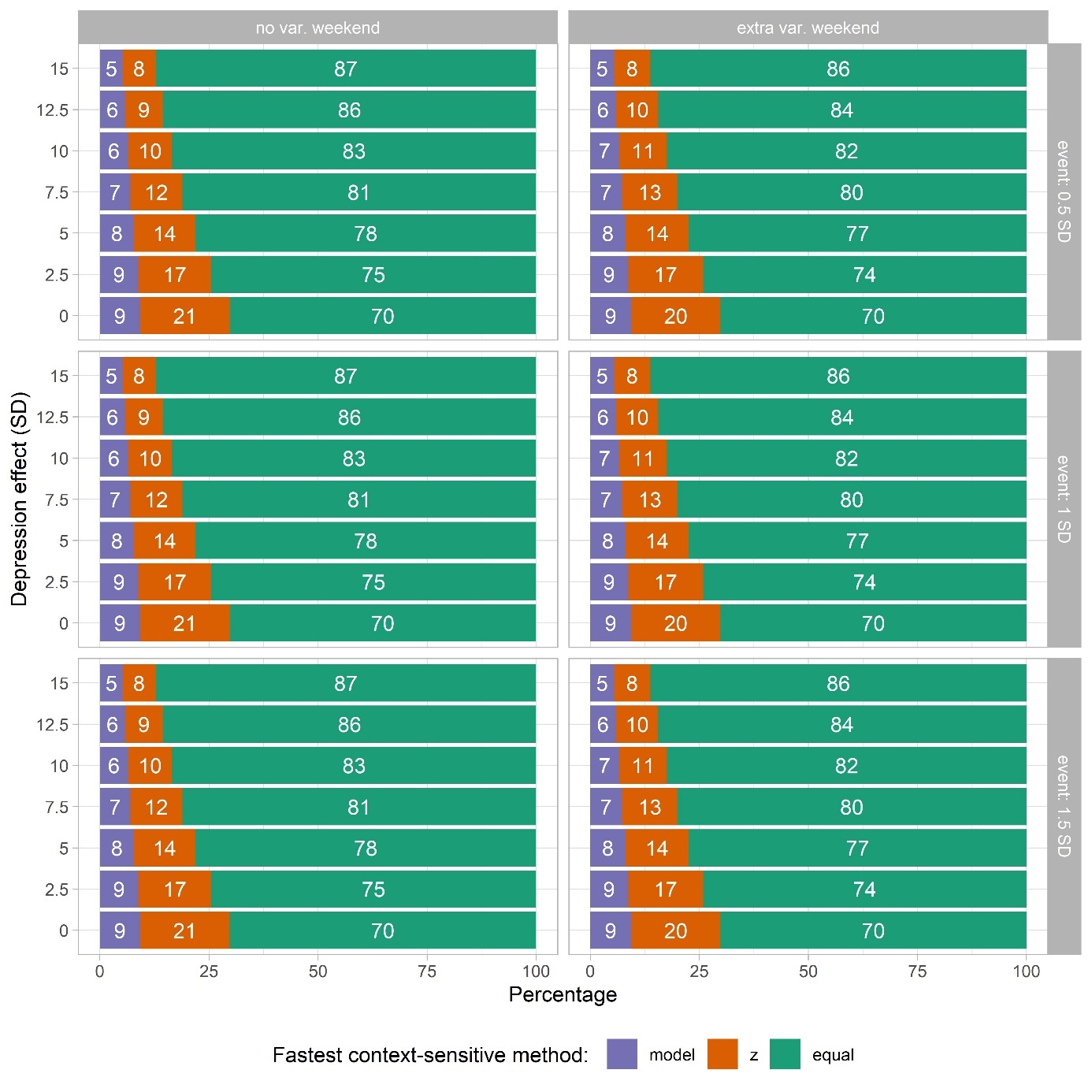


*Fig. S4. The percentage of replicates for which context-sensitive methods yielded a warning sign equally quick (equal) or not (model, z), assuming moderate weekend effects (0.4 SD), no bias in the detection of events, and an event frequency of 1/3.* The z method consistently yields faster warnings as compared to the modeling method. The difference between methods is most apparent in case of small depression effects, and hardly changes when adding weekend variability. This is noteworthy, as time-varying variability is the feature that can theoretically be handled by the z method, but not the modeling method.

Table S1 below describes the effect of contextual factors on mental states, as well as the performance of different SPC methods, in empirical data. Estimates were obtained by fitting two linear regression models for each mental state separately. First, we inspected the standardized effect of weekends and events by (1) computing day-means for each mental state (Schat, Tuerlinckx, Smit, et al., 2023), (2) standardizing the day-means, and (3) fitting linear models with the standardized day-means as outcome and weekends and events as predictors. Here, events were dichotomized, meaning that a day-mean below 0 was considered indicative of a negative event (1) while a day-mean above 0 was not (0) (Lachowicz et al., 2023; Li et al., 2024). In a second model, we inspected the effect of depression status on mean levels of mental states by fitting a linear model with standardized day-means as outcome and depression status (0 = before transition towards depression, 1 = after transition towards depression) as a predictor. Results indicate that the effects in the empirical data (average event = 0.63 SD, average depression = 0.31 SD) overlap with those in the simulation study, with the exception of weekend effects (average = 0.02 SD), which were simulated as more negative.

| *Table S1. Effects of contextual factors (weekend, event) and depression status on negative mental states and the earliest warning signs detected through different statistical process control methods* | | | | | | | | | |
| --- | --- | --- | --- | --- | --- | --- | --- | --- | --- |
|  |  |  |  |  |  |  | *SPC methods* | | |
| item | weekend | P | event | P | depr. | P | standard | z | model |
| restless | -0.02 | 0.78 | 0.63 | 0.00 | 0.33 | 0.00 | 51 | 51 | 51 |
| worry | 0.17 | 0.06 | 0.68 | 0.00 | 0.41 | 0.00 | 52 | 52 | 52 |
| ashamed | 0.02 | 0.82 | 0.64 | 0.00 | 0.19 | 0.01 | 52 | 52 | 52 |
| agitated | -0.05 | 0.52 | 0.77 | 0.00 | 0.28 | 0.00 | 53 | 51 | 51 |
| self-doubt | 0.06 | 0.47 | 0.76 | 0.00 | 0.10 | 0.23 | 54 | 52 | 54 |
| doubt | -0.02 | 0.83 | 0.66 | 0.00 | 0.08 | 0.34 | 76 | 75 | 58 |
| irritated | -0.13 | 0.06 | 0.68 | 0.00 | 0.24 | 0.00 | 77 | 76 | 76 |
| suspicious | 0.13 | 0.14 | 0.75 | 0.00 | 0.67 | 0.00 | 77 | 52 | 77 |
| guilty | 0.06 | 0.42 | 0.55 | 0.00 | 0.31 | 0.00 | 90 | 89 | 92 |
| down | 0.11 | 0.25 | 0.67 | 0.00 | 0.42 | 0.00 | 91 | 90 | 92 |
| lonely | 0.03 | 0.74 | 0.48 | 0.00 | 0.35 | 0.00 | - | 52 | - |
| anxious | -0.06 | 0.48 | 0.29 | 0.00 | 0.31 | 0.00 | - | - | - |
|  | | | | | | | | | |

| *Table S2. Average run length differences between context-sensitive SPC methods (model, z) and the benchmark methods. Negative values indicate that context-sensitive SPC methods yielded an earlier warning sign as compared to the benchmark method.* | | | | | | | | |
| --- | --- | --- | --- | --- | --- | --- | --- | --- |
| Depression effect (SD) | Weekend effect (SD) | Event effect (SD) | Extra weekend variability | Event frequency | Model |  | Z |  |
|  |  |  |  |  | Mean | SD | Mean | SD |
| 0 | 0.15 | 0.50 | 0 | 1/3 | -16,02 | 521,47 | -52,77 | 517,44 |
| 0 | 0.15 | 0.50 | 0 | 1/5 | -17,23 | 488,76 | -53,44 | 496,54 |
| 0 | 0.15 | 0.50 | 1 | 1/3 | -16,45 | 498,18 | -50,46 | 504,87 |
| 0 | 0.15 | 0.50 | 1 | 1/5 | -15,52 | 469,87 | -50,88 | 486,01 |
| 0 | 0.15 | 1.00 | 0 | 1/3 | -5,14 | 656,86 | -41,89 | 632,53 |
| 0 | 0.15 | 1.00 | 0 | 1/5 | 1,65 | 623,62 | -34,57 | 602,17 |
| 0 | 0.15 | 1.00 | 1 | 1/3 | -7,44 | 638,02 | -41,50 | 620,20 |
| 0 | 0.15 | 1.00 | 1 | 1/5 | -0,82 | 607,34 | -36,18 | 586,07 |
| 0 | 0.15 | 1.50 | 0 | 1/3 | 11,89 | 725,02 | -24,85 | 691,89 |
| 0 | 0.15 | 1.50 | 0 | 1/5 | 32,61 | 684,43 | -3,60 | 655,90 |
| 0 | 0.15 | 1.50 | 1 | 1/3 | 9,85 | 710,44 | -24,26 | 683,72 |
| 0 | 0.15 | 1.50 | 1 | 1/5 | 25,40 | 676,84 | -10,02 | 646,63 |
| 0 | 0.40 | 0.50 | 0 | 1/3 | -41,05 | 554,71 | -77,80 | 557,60 |
| 0 | 0.40 | 0.50 | 0 | 1/5 | -42,58 | 516,03 | -78,72 | 527,75 |
| 0 | 0.40 | 0.50 | 1 | 1/3 | -37,01 | 529,06 | -71,01 | 534,78 |
| 0 | 0.40 | 0.50 | 1 | 1/5 | -35,68 | 497,46 | -70,93 | 517,96 |
| 0 | 0.40 | 1.00 | 0 | 1/3 | -26,22 | 681,60 | -62,96 | 661,02 |
| 0 | 0.40 | 1.00 | 0 | 1/5 | -19,02 | 643,51 | -55,16 | 626,30 |
| 0 | 0.40 | 1.00 | 1 | 1/3 | -23,77 | 652,04 | -57,81 | 637,27 |
| 0 | 0.40 | 1.00 | 1 | 1/5 | -19,26 | 623,53 | -54,51 | 606,82 |
| 0 | 0.40 | 1.50 | 0 | 1/3 | -4,01 | 743,69 | -40,76 | 712,45 |
| 0 | 0.40 | 1.50 | 0 | 1/5 | 16,31 | 698,70 | -19,83 | 672,73 |
| 0 | 0.40 | 1.50 | 1 | 1/3 | -5,09 | 720,86 | -39,19 | 696,09 |
| 0 | 0.40 | 1.50 | 1 | 1/5 | 11,51 | 689,13 | -23,80 | 661,81 |
| 0 | 0.65 | 0.50 | 0 | 1/3 | -89,20 | 611,37 | -125,94 | 624,40 |
| 0 | 0.65 | 0.50 | 0 | 1/5 | -93,22 | 583,95 | -129,32 | 600,74 |
| 0 | 0.65 | 0.50 | 1 | 1/3 | -76,48 | 579,92 | -110,59 | 591,79 |
| 0 | 0.65 | 0.50 | 1 | 1/5 | -76,06 | 554,15 | -111,50 | 574,51 |
| 0 | 0.65 | 1.00 | 0 | 1/3 | -66,28 | 722,76 | -103,02 | 707,75 |
| 0 | 0.65 | 1.00 | 0 | 1/5 | -61,37 | 682,05 | -97,47 | 671,70 |
| 0 | 0.65 | 1.00 | 1 | 1/3 | -57,61 | 688,63 | -91,75 | 678,03 |
| 0 | 0.65 | 1.00 | 1 | 1/5 | -53,05 | 657,00 | -88,44 | 646,61 |
| 0 | 0.65 | 1.50 | 0 | 1/3 | -35,19 | 775,89 | -71,92 | 747,61 |
| 0 | 0.65 | 1.50 | 0 | 1/5 | -17,01 | 731,22 | -53,10 | 708,95 |
| 0 | 0.65 | 1.50 | 1 | 1/3 | -32,04 | 747,05 | -66,24 | 725,21 |
| 0 | 0.65 | 1.50 | 1 | 1/5 | -16,34 | 717,58 | -51,76 | 693,14 |
| 2.5 | 0.15 | 0.50 | 0 | 1/3 | -3,70 | 48,60 | -5,97 | 49,70 |
| 2.5 | 0.15 | 0.50 | 0 | 1/5 | -2,95 | 44,37 | -5,41 | 46,24 |
| 2.5 | 0.15 | 0.50 | 1 | 1/3 | -3,64 | 55,89 | -6,67 | 57,13 |
| 2.5 | 0.15 | 0.50 | 1 | 1/5 | -3,31 | 52,15 | -6,42 | 54,85 |
| 2.5 | 0.15 | 1.00 | 0 | 1/3 | -10,58 | 69,72 | -12,85 | 69,45 |
| 2.5 | 0.15 | 1.00 | 0 | 1/5 | -7,69 | 63,49 | -10,15 | 63,32 |
| 2.5 | 0.15 | 1.00 | 1 | 1/3 | -10,18 | 79,69 | -13,22 | 79,33 |
| 2.5 | 0.15 | 1.00 | 1 | 1/5 | -7,65 | 73,74 | -10,76 | 73,70 |
| 2.5 | 0.15 | 1.50 | 0 | 1/3 | -20,04 | 87,54 | -22,32 | 86,94 |
| 2.5 | 0.15 | 1.50 | 0 | 1/5 | -14,61 | 80,67 | -17,07 | 80,20 |
| 2.5 | 0.15 | 1.50 | 1 | 1/3 | -18,52 | 99,30 | -21,55 | 98,54 |
| 2.5 | 0.15 | 1.50 | 1 | 1/5 | -13,23 | 90,90 | -16,35 | 90,45 |
| 2.5 | 0.40 | 0.50 | 0 | 1/3 | -5,46 | 50,79 | -7,73 | 52,23 |
| 2.5 | 0.40 | 0.50 | 0 | 1/5 | -4,63 | 47,48 | -7,09 | 49,25 |
| 2.5 | 0.40 | 0.50 | 1 | 1/3 | -5,44 | 58,82 | -8,49 | 60,21 |
| 2.5 | 0.40 | 0.50 | 1 | 1/5 | -4,98 | 54,53 | -8,09 | 57,42 |
| 2.5 | 0.40 | 1.00 | 0 | 1/3 | -12,40 | 71,89 | -14,67 | 71,77 |
| 2.5 | 0.40 | 1.00 | 0 | 1/5 | -9,46 | 65,71 | -11,92 | 65,64 |
| 2.5 | 0.40 | 1.00 | 1 | 1/3 | -11,83 | 81,27 | -14,87 | 81,11 |
| 2.5 | 0.40 | 1.00 | 1 | 1/5 | -9,22 | 75,78 | -12,33 | 75,64 |
| 2.5 | 0.40 | 1.50 | 0 | 1/3 | -21,55 | 89,51 | -23,83 | 89,01 |
| 2.5 | 0.40 | 1.50 | 0 | 1/5 | -16,16 | 82,24 | -18,62 | 81,85 |
| 2.5 | 0.40 | 1.50 | 1 | 1/3 | -20,21 | 101,46 | -23,25 | 100,86 |
| 2.5 | 0.40 | 1.50 | 1 | 1/5 | -14,87 | 92,87 | -17,98 | 92,28 |
| 2.5 | 0.65 | 0.50 | 0 | 1/3 | -9,19 | 55,92 | -11,46 | 57,58 |
| 2.5 | 0.65 | 0.50 | 0 | 1/5 | -8,43 | 53,08 | -10,89 | 54,84 |
| 2.5 | 0.65 | 0.50 | 1 | 1/3 | -9,05 | 63,65 | -12,09 | 65,24 |
| 2.5 | 0.65 | 0.50 | 1 | 1/5 | -8,58 | 60,13 | -11,70 | 62,84 |
| 2.5 | 0.65 | 1.00 | 0 | 1/3 | -16,17 | 76,68 | -18,45 | 76,84 |
| 2.5 | 0.65 | 1.00 | 0 | 1/5 | -13,06 | 70,08 | -15,52 | 70,07 |
| 2.5 | 0.65 | 1.00 | 1 | 1/3 | -15,34 | 85,92 | -18,39 | 85,97 |
| 2.5 | 0.65 | 1.00 | 1 | 1/5 | -12,80 | 80,26 | -15,92 | 80,48 |
| 2.5 | 0.65 | 1.50 | 0 | 1/3 | -25,01 | 94,20 | -27,29 | 93,88 |
| 2.5 | 0.65 | 1.50 | 0 | 1/5 | -19,57 | 86,11 | -22,04 | 85,85 |
| 2.5 | 0.65 | 1.50 | 1 | 1/3 | -23,64 | 106,07 | -26,68 | 105,58 |
| 2.5 | 0.65 | 1.50 | 1 | 1/5 | -18,40 | 96,84 | -21,52 | 96,34 |
| 5 | 0.15 | 0.50 | 0 | 1/3 | -1,09 | 10,44 | -1,54 | 10,88 |
| 5 | 0.15 | 0.50 | 0 | 1/5 | -0,78 | 9,66 | -1,26 | 10,21 |
| 5 | 0.15 | 0.50 | 1 | 1/3 | -1,15 | 12,78 | -1,74 | 13,40 |
| 5 | 0.15 | 0.50 | 1 | 1/5 | -0,91 | 12,04 | -1,51 | 12,72 |
| 5 | 0.15 | 1.00 | 0 | 1/3 | -3,94 | 16,50 | -4,39 | 16,63 |
| 5 | 0.15 | 1.00 | 0 | 1/5 | -2,85 | 14,89 | -3,33 | 15,05 |
| 5 | 0.15 | 1.00 | 1 | 1/3 | -3,88 | 19,55 | -4,47 | 19,65 |
| 5 | 0.15 | 1.00 | 1 | 1/5 | -2,87 | 17,62 | -3,47 | 17,79 |
| 5 | 0.15 | 1.50 | 0 | 1/3 | -8,35 | 23,08 | -8,81 | 23,17 |
| 5 | 0.15 | 1.50 | 0 | 1/5 | -6,14 | 20,30 | -6,62 | 20,34 |
| 5 | 0.15 | 1.50 | 1 | 1/3 | -8,05 | 26,31 | -8,63 | 26,30 |
| 5 | 0.15 | 1.50 | 1 | 1/5 | -6,09 | 23,69 | -6,69 | 23,67 |
| 5 | 0.40 | 0.50 | 0 | 1/3 | -1,26 | 10,95 | -1,71 | 11,38 |
| 5 | 0.40 | 0.50 | 0 | 1/5 | -0,93 | 10,14 | -1,41 | 10,63 |
| 5 | 0.40 | 0.50 | 1 | 1/3 | -1,35 | 13,29 | -1,94 | 13,88 |
| 5 | 0.40 | 0.50 | 1 | 1/5 | -1,14 | 12,53 | -1,74 | 13,18 |
| 5 | 0.40 | 1.00 | 0 | 1/3 | -4,14 | 16,87 | -4,59 | 17,02 |
| 5 | 0.40 | 1.00 | 0 | 1/5 | -3,04 | 15,26 | -3,52 | 15,44 |
| 5 | 0.40 | 1.00 | 1 | 1/3 | -4,09 | 19,85 | -4,68 | 19,98 |
| 5 | 0.40 | 1.00 | 1 | 1/5 | -3,09 | 18,10 | -3,68 | 18,32 |
| 5 | 0.40 | 1.50 | 0 | 1/3 | -8,57 | 23,53 | -9,03 | 23,62 |
| 5 | 0.40 | 1.50 | 0 | 1/5 | -6,35 | 20,85 | -6,83 | 20,91 |
| 5 | 0.40 | 1.50 | 1 | 1/3 | -8,28 | 26,69 | -8,87 | 26,70 |
| 5 | 0.40 | 1.50 | 1 | 1/5 | -6,30 | 24,05 | -6,89 | 24,06 |
| 5 | 0.65 | 0.50 | 0 | 1/3 | -1,85 | 11,92 | -2,30 | 12,31 |
| 5 | 0.65 | 0.50 | 0 | 1/5 | -1,53 | 10,98 | -2,01 | 11,43 |
| 5 | 0.65 | 0.50 | 1 | 1/3 | -1,97 | 14,26 | -2,56 | 14,87 |
| 5 | 0.65 | 0.50 | 1 | 1/5 | -1,75 | 13,53 | -2,34 | 14,18 |
| 5 | 0.65 | 1.00 | 0 | 1/3 | -4,76 | 17,65 | -5,21 | 17,81 |
| 5 | 0.65 | 1.00 | 0 | 1/5 | -3,65 | 16,05 | -4,13 | 16,20 |
| 5 | 0.65 | 1.00 | 1 | 1/3 | -4,74 | 20,71 | -5,33 | 20,85 |
| 5 | 0.65 | 1.00 | 1 | 1/5 | -3,76 | 18,89 | -4,35 | 19,06 |
| 5 | 0.65 | 1.50 | 0 | 1/3 | -9,22 | 24,40 | -9,67 | 24,50 |
| 5 | 0.65 | 1.50 | 0 | 1/5 | -6,99 | 21,81 | -7,47 | 21,86 |
| 5 | 0.65 | 1.50 | 1 | 1/3 | -9,00 | 27,71 | -9,58 | 27,73 |
| 5 | 0.65 | 1.50 | 1 | 1/5 | -6,94 | 24,73 | -7,54 | 24,75 |
| 7.5 | 0.15 | 0.50 | 0 | 1/3 | -0,45 | 3,84 | -0,63 | 4,02 |
| 7.5 | 0.15 | 0.50 | 0 | 1/5 | -0,31 | 3,55 | -0,48 | 3,75 |
| 7.5 | 0.15 | 0.50 | 1 | 1/3 | -0,42 | 4,78 | -0,64 | 4,96 |
| 7.5 | 0.15 | 0.50 | 1 | 1/5 | -0,31 | 4,39 | -0,54 | 4,69 |
| 7.5 | 0.15 | 1.00 | 0 | 1/3 | -1,89 | 6,34 | -2,06 | 6,41 |
| 7.5 | 0.15 | 1.00 | 0 | 1/5 | -1,39 | 5,74 | -1,55 | 5,79 |
| 7.5 | 0.15 | 1.00 | 1 | 1/3 | -1,81 | 7,61 | -2,03 | 7,65 |
| 7.5 | 0.15 | 1.00 | 1 | 1/5 | -1,33 | 6,82 | -1,56 | 6,93 |
| 7.5 | 0.15 | 1.50 | 0 | 1/3 | -4,18 | 9,32 | -4,35 | 9,36 |
| 7.5 | 0.15 | 1.50 | 0 | 1/5 | -3,13 | 8,19 | -3,30 | 8,23 |
| 7.5 | 0.15 | 1.50 | 1 | 1/3 | -4,07 | 10,81 | -4,29 | 10,82 |
| 7.5 | 0.15 | 1.50 | 1 | 1/5 | -3,02 | 9,51 | -3,24 | 9,54 |
| 7.5 | 0.40 | 0.50 | 0 | 1/3 | -0,43 | 4,01 | -0,61 | 4,17 |
| 7.5 | 0.40 | 0.50 | 0 | 1/5 | -0,28 | 3,73 | -0,45 | 3,90 |
| 7.5 | 0.40 | 0.50 | 1 | 1/3 | -0,39 | 4,96 | -0,61 | 5,12 |
| 7.5 | 0.40 | 0.50 | 1 | 1/5 | -0,28 | 4,52 | -0,51 | 4,80 |
| 7.5 | 0.40 | 1.00 | 0 | 1/3 | -1,85 | 6,43 | -2,02 | 6,50 |
| 7.5 | 0.40 | 1.00 | 0 | 1/5 | -1,35 | 5,82 | -1,51 | 5,87 |
| 7.5 | 0.40 | 1.00 | 1 | 1/3 | -1,78 | 7,75 | -2,00 | 7,78 |
| 7.5 | 0.40 | 1.00 | 1 | 1/5 | -1,31 | 6,96 | -1,54 | 7,06 |
| 7.5 | 0.40 | 1.50 | 0 | 1/3 | -4,18 | 9,49 | -4,36 | 9,54 |
| 7.5 | 0.40 | 1.50 | 0 | 1/5 | -3,10 | 8,28 | -3,27 | 8,31 |
| 7.5 | 0.40 | 1.50 | 1 | 1/3 | -4,06 | 10,89 | -4,28 | 10,90 |
| 7.5 | 0.40 | 1.50 | 1 | 1/5 | -3,02 | 9,66 | -3,24 | 9,70 |
| 7.5 | 0.65 | 0.50 | 0 | 1/3 | -0,57 | 4,27 | -0,74 | 4,43 |
| 7.5 | 0.65 | 0.50 | 0 | 1/5 | -0,43 | 4,02 | -0,60 | 4,17 |
| 7.5 | 0.65 | 0.50 | 1 | 1/3 | -0,56 | 5,26 | -0,78 | 5,42 |
| 7.5 | 0.65 | 0.50 | 1 | 1/5 | -0,44 | 4,89 | -0,66 | 5,13 |
| 7.5 | 0.65 | 1.00 | 0 | 1/3 | -1,99 | 6,63 | -2,17 | 6,70 |
| 7.5 | 0.65 | 1.00 | 0 | 1/5 | -1,50 | 6,05 | -1,67 | 6,11 |
| 7.5 | 0.65 | 1.00 | 1 | 1/3 | -1,94 | 7,97 | -2,16 | 8,00 |
| 7.5 | 0.65 | 1.00 | 1 | 1/5 | -1,47 | 7,17 | -1,69 | 7,27 |
| 7.5 | 0.65 | 1.50 | 0 | 1/3 | -4,34 | 9,71 | -4,52 | 9,76 |
| 7.5 | 0.65 | 1.50 | 0 | 1/5 | -3,25 | 8,50 | -3,42 | 8,53 |
| 7.5 | 0.65 | 1.50 | 1 | 1/3 | -4,22 | 11,16 | -4,44 | 11,18 |
| 7.5 | 0.65 | 1.50 | 1 | 1/5 | -3,18 | 9,90 | -3,40 | 9,95 |
| 10 | 0.15 | 0.50 | 0 | 1/3 | -0,24 | 1,99 | -0,32 | 2,07 |
| 10 | 0.15 | 0.50 | 0 | 1/5 | -0,16 | 1,84 | -0,25 | 1,95 |
| 10 | 0.15 | 0.50 | 1 | 1/3 | -0,24 | 2,38 | -0,36 | 2,53 |
| 10 | 0.15 | 0.50 | 1 | 1/5 | -0,16 | 2,19 | -0,27 | 2,35 |
| 10 | 0.15 | 1.00 | 0 | 1/3 | -1,14 | 3,31 | -1,22 | 3,35 |
| 10 | 0.15 | 1.00 | 0 | 1/5 | -0,82 | 2,91 | -0,91 | 2,96 |
| 10 | 0.15 | 1.00 | 1 | 1/3 | -1,11 | 3,88 | -1,22 | 3,95 |
| 10 | 0.15 | 1.00 | 1 | 1/5 | -0,79 | 3,45 | -0,90 | 3,50 |
| 10 | 0.15 | 1.50 | 0 | 1/3 | -2,59 | 4,95 | -2,67 | 4,97 |
| 10 | 0.15 | 1.50 | 0 | 1/5 | -1,92 | 4,26 | -2,00 | 4,28 |
| 10 | 0.15 | 1.50 | 1 | 1/3 | -2,49 | 5,60 | -2,60 | 5,64 |
| 10 | 0.15 | 1.50 | 1 | 1/5 | -1,83 | 4,88 | -1,94 | 4,91 |
| 10 | 0.40 | 0.50 | 0 | 1/3 | -0,17 | 2,05 | -0,25 | 2,13 |
| 10 | 0.40 | 0.50 | 0 | 1/5 | -0,08 | 1,93 | -0,17 | 2,02 |
| 10 | 0.40 | 0.50 | 1 | 1/3 | -0,17 | 2,47 | -0,29 | 2,59 |
| 10 | 0.40 | 0.50 | 1 | 1/5 | -0,10 | 2,29 | -0,21 | 2,42 |
| 10 | 0.40 | 1.00 | 0 | 1/3 | -1,07 | 3,35 | -1,15 | 3,38 |
| 10 | 0.40 | 1.00 | 0 | 1/5 | -0,75 | 2,95 | -0,84 | 2,99 |
| 10 | 0.40 | 1.00 | 1 | 1/3 | -1,03 | 3,93 | -1,15 | 3,98 |
| 10 | 0.40 | 1.00 | 1 | 1/5 | -0,73 | 3,48 | -0,84 | 3,54 |
| 10 | 0.40 | 1.50 | 0 | 1/3 | -2,52 | 4,99 | -2,60 | 5,01 |
| 10 | 0.40 | 1.50 | 0 | 1/5 | -1,86 | 4,29 | -1,94 | 4,31 |
| 10 | 0.40 | 1.50 | 1 | 1/3 | -2,43 | 5,64 | -2,54 | 5,68 |
| 10 | 0.40 | 1.50 | 1 | 1/5 | -1,77 | 4,90 | -1,87 | 4,93 |
| 10 | 0.65 | 0.50 | 0 | 1/3 | -0,20 | 2,20 | -0,28 | 2,27 |
| 10 | 0.65 | 0.50 | 0 | 1/5 | -0,11 | 2,08 | -0,20 | 2,16 |
| 10 | 0.65 | 0.50 | 1 | 1/3 | -0,20 | 2,62 | -0,32 | 2,73 |
| 10 | 0.65 | 0.50 | 1 | 1/5 | -0,13 | 2,45 | -0,24 | 2,56 |
| 10 | 0.65 | 1.00 | 0 | 1/3 | -1,11 | 3,45 | -1,19 | 3,49 |
| 10 | 0.65 | 1.00 | 0 | 1/5 | -0,78 | 3,06 | -0,87 | 3,09 |
| 10 | 0.65 | 1.00 | 1 | 1/3 | -1,06 | 4,02 | -1,17 | 4,07 |
| 10 | 0.65 | 1.00 | 1 | 1/5 | -0,76 | 3,60 | -0,86 | 3,65 |
| 10 | 0.65 | 1.50 | 0 | 1/3 | -2,56 | 5,09 | -2,64 | 5,11 |
| 10 | 0.65 | 1.50 | 0 | 1/5 | -1,89 | 4,39 | -1,97 | 4,41 |
| 10 | 0.65 | 1.50 | 1 | 1/3 | -2,46 | 5,74 | -2,57 | 5,77 |
| 10 | 0.65 | 1.50 | 1 | 1/5 | -1,80 | 5,01 | -1,91 | 5,04 |
| 12.5 | 0.15 | 0.50 | 0 | 1/3 | -0,14 | 1,25 | -0,19 | 1,31 |
| 12.5 | 0.15 | 0.50 | 0 | 1/5 | -0,09 | 1,12 | -0,14 | 1,20 |
| 12.5 | 0.15 | 0.50 | 1 | 1/3 | -0,15 | 1,46 | -0,22 | 1,57 |
| 12.5 | 0.15 | 0.50 | 1 | 1/5 | -0,09 | 1,36 | -0,17 | 1,45 |
| 12.5 | 0.15 | 1.00 | 0 | 1/3 | -0,77 | 2,07 | -0,82 | 2,09 |
| 12.5 | 0.15 | 1.00 | 0 | 1/5 | -0,55 | 1,81 | -0,60 | 1,85 |
| 12.5 | 0.15 | 1.00 | 1 | 1/3 | -0,76 | 2,39 | -0,83 | 2,44 |
| 12.5 | 0.15 | 1.00 | 1 | 1/5 | -0,54 | 2,14 | -0,61 | 2,17 |
| 12.5 | 0.15 | 1.50 | 0 | 1/3 | -1,79 | 3,07 | -1,84 | 3,08 |
| 12.5 | 0.15 | 1.50 | 0 | 1/5 | -1,31 | 2,63 | -1,35 | 2,65 |
| 12.5 | 0.15 | 1.50 | 1 | 1/3 | -1,73 | 3,43 | -1,80 | 3,46 |
| 12.5 | 0.15 | 1.50 | 1 | 1/5 | -1,27 | 3,00 | -1,34 | 3,02 |
| 12.5 | 0.40 | 0.50 | 0 | 1/3 | -0,06 | 1,29 | -0,10 | 1,34 |
| 12.5 | 0.40 | 0.50 | 0 | 1/5 | 0,00 | 1,18 | -0,05 | 1,24 |
| 12.5 | 0.40 | 0.50 | 1 | 1/3 | -0,06 | 1,51 | -0,13 | 1,60 |
| 12.5 | 0.40 | 0.50 | 1 | 1/5 | -0,01 | 1,43 | -0,08 | 1,50 |
| 12.5 | 0.40 | 1.00 | 0 | 1/3 | -0,69 | 2,09 | -0,73 | 2,11 |
| 12.5 | 0.40 | 1.00 | 0 | 1/5 | -0,46 | 1,83 | -0,51 | 1,86 |
| 12.5 | 0.40 | 1.00 | 1 | 1/3 | -0,68 | 2,41 | -0,75 | 2,45 |
| 12.5 | 0.40 | 1.00 | 1 | 1/5 | -0,46 | 2,15 | -0,53 | 2,19 |
| 12.5 | 0.40 | 1.50 | 0 | 1/3 | -1,71 | 3,09 | -1,76 | 3,11 |
| 12.5 | 0.40 | 1.50 | 0 | 1/5 | -1,22 | 2,64 | -1,27 | 2,67 |
| 12.5 | 0.40 | 1.50 | 1 | 1/3 | -1,65 | 3,46 | -1,72 | 3,49 |
| 12.5 | 0.40 | 1.50 | 1 | 1/5 | -1,20 | 3,02 | -1,27 | 3,03 |
| 12.5 | 0.65 | 0.50 | 0 | 1/3 | -0,04 | 1,37 | -0,08 | 1,42 |
| 12.5 | 0.65 | 0.50 | 0 | 1/5 | 0,02 | 1,27 | -0,03 | 1,32 |
| 12.5 | 0.65 | 0.50 | 1 | 1/3 | -0,05 | 1,62 | -0,12 | 1,70 |
| 12.5 | 0.65 | 0.50 | 1 | 1/5 | 0,00 | 1,52 | -0,07 | 1,59 |
| 12.5 | 0.65 | 1.00 | 0 | 1/3 | -0,68 | 2,15 | -0,72 | 2,17 |
| 12.5 | 0.65 | 1.00 | 0 | 1/5 | -0,44 | 1,89 | -0,49 | 1,92 |
| 12.5 | 0.65 | 1.00 | 1 | 1/3 | -0,66 | 2,47 | -0,73 | 2,51 |
| 12.5 | 0.65 | 1.00 | 1 | 1/5 | -0,44 | 2,21 | -0,51 | 2,24 |
| 12.5 | 0.65 | 1.50 | 0 | 1/3 | -1,70 | 3,15 | -1,75 | 3,16 |
| 12.5 | 0.65 | 1.50 | 0 | 1/5 | -1,22 | 2,70 | -1,26 | 2,72 |
| 12.5 | 0.65 | 1.50 | 1 | 1/3 | -1,65 | 3,52 | -1,72 | 3,55 |
| 12.5 | 0.65 | 1.50 | 1 | 1/5 | -1,18 | 3,07 | -1,25 | 3,08 |
| 15 | 0.15 | 0.50 | 0 | 1/3 | -0,09 | 0,87 | -0,12 | 0,92 |
| 15 | 0.15 | 0.50 | 0 | 1/5 | -0,04 | 0,80 | -0,08 | 0,85 |
| 15 | 0.15 | 0.50 | 1 | 1/3 | -0,09 | 1,02 | -0,14 | 1,09 |
| 15 | 0.15 | 0.50 | 1 | 1/5 | -0,05 | 0,93 | -0,09 | 1,00 |
| 15 | 0.15 | 1.00 | 0 | 1/3 | -0,57 | 1,45 | -0,60 | 1,47 |
| 15 | 0.15 | 1.00 | 0 | 1/5 | -0,39 | 1,27 | -0,43 | 1,28 |
| 15 | 0.15 | 1.00 | 1 | 1/3 | -0,54 | 1,62 | -0,58 | 1,65 |
| 15 | 0.15 | 1.00 | 1 | 1/5 | -0,38 | 1,44 | -0,42 | 1,47 |
| 15 | 0.15 | 1.50 | 0 | 1/3 | -1,34 | 2,13 | -1,37 | 2,14 |
| 15 | 0.15 | 1.50 | 0 | 1/5 | -0,97 | 1,83 | -1,01 | 1,84 |
| 15 | 0.15 | 1.50 | 1 | 1/3 | -1,28 | 2,36 | -1,32 | 2,38 |
| 15 | 0.15 | 1.50 | 1 | 1/5 | -0,93 | 2,05 | -0,97 | 2,06 |
| 15 | 0.40 | 0.50 | 0 | 1/3 | 0,01 | 0,89 | -0,02 | 0,94 |
| 15 | 0.40 | 0.50 | 0 | 1/5 | 0,06 | 0,83 | 0,02 | 0,87 |
| 15 | 0.40 | 0.50 | 1 | 1/3 | 0,01 | 1,05 | -0,04 | 1,11 |
| 15 | 0.40 | 0.50 | 1 | 1/5 | 0,05 | 0,98 | 0,01 | 1,04 |
| 15 | 0.40 | 1.00 | 0 | 1/3 | -0,47 | 1,47 | -0,50 | 1,49 |
| 15 | 0.40 | 1.00 | 0 | 1/5 | -0,30 | 1,28 | -0,33 | 1,30 |
| 15 | 0.40 | 1.00 | 1 | 1/3 | -0,45 | 1,64 | -0,49 | 1,67 |
| 15 | 0.40 | 1.00 | 1 | 1/5 | -0,29 | 1,46 | -0,33 | 1,48 |
| 15 | 0.40 | 1.50 | 0 | 1/3 | -1,25 | 2,15 | -1,28 | 2,16 |
| 15 | 0.40 | 1.50 | 0 | 1/5 | -0,88 | 1,84 | -0,91 | 1,84 |
| 15 | 0.40 | 1.50 | 1 | 1/3 | -1,19 | 2,38 | -1,24 | 2,40 |
| 15 | 0.40 | 1.50 | 1 | 1/5 | -0,85 | 2,06 | -0,89 | 2,08 |
| 15 | 0.65 | 0.50 | 0 | 1/3 | 0,05 | 0,95 | 0,02 | 0,99 |
| 15 | 0.65 | 0.50 | 0 | 1/5 | 0,09 | 0,89 | 0,06 | 0,92 |
| 15 | 0.65 | 0.50 | 1 | 1/3 | 0,05 | 1,11 | 0,00 | 1,16 |
| 15 | 0.65 | 0.50 | 1 | 1/5 | 0,09 | 1,05 | 0,05 | 1,10 |
| 15 | 0.65 | 1.00 | 0 | 1/3 | -0,43 | 1,50 | -0,47 | 1,51 |
| 15 | 0.65 | 1.00 | 0 | 1/5 | -0,26 | 1,31 | -0,29 | 1,33 |
| 15 | 0.65 | 1.00 | 1 | 1/3 | -0,41 | 1,68 | -0,46 | 1,71 |
| 15 | 0.65 | 1.00 | 1 | 1/5 | -0,25 | 1,51 | -0,29 | 1,53 |
| 15 | 0.65 | 1.50 | 0 | 1/3 | -1,21 | 2,18 | -1,24 | 2,19 |
| 15 | 0.65 | 1.50 | 0 | 1/5 | -0,84 | 1,87 | -0,88 | 1,88 |
| 15 | 0.65 | 1.50 | 1 | 1/3 | -1,16 | 2,42 | -1,21 | 2,44 |
| 15 | 0.65 | 1.50 | 1 | 1/5 | -0,81 | 2,10 | -0,85 | 2,11 |
| *Note.* For readability, results are limited to the situation in which there is no bias in event detection. Results stratified based on different bias levels are available upon request. | | | | | | | | |
